# Spadin, a Sortilin-Derived Peptide, Targeting Rodent TREK-1 Channels: A New Concept in the Antidepressant Drug Design

**DOI:** 10.1371/journal.pbio.1000355

**Published:** 2010-04-13

**Authors:** Jean Mazella, Olivier Pétrault, Guillaume Lucas, Emmanuel Deval, Sophie Béraud-Dufour, Carine Gandin, Malika El-Yacoubi, Catherine Widmann, Alice Guyon, Eric Chevet, Said Taouji, Grégory Conductier, Alain Corinus, Thierry Coppola, Gabriella Gobbi, Jean-Louis Nahon, Catherine Heurteaux, Marc Borsotto

**Affiliations:** 1 Institut de Pharmacologie Moléculaire et Cellulaire, Centre National de la Recherche Scientifique (CNRS), Université de Nice Sophia Antipolis, Valbonne, France; 2 Department of Psychiatry, Centre de Recherche Fernand Seguin Université de Montréal, Montréal, Québec, Canada; 3 Laboratoire de Neuropharmacologie, Faculté de Pharmacie, CNRS, Université de Lyon 1, Lyon, France; 4 Avenir, Inserm U889, Université Victor Segalen Bordeaux 2, Bordeaux, France; 5 Department of Psychiatry, McGill University, Montréal, Québec, Canada; Mount Sinai School of Medicine, United States of America

## Abstract

Current antidepressant treatments are inadequate for many individuals, and when they are effective, they require several weeks of administration before a therapeutic effect can be observed. Improving the treatment of depression is challenging. Recently, the two-pore domain potassium channel TREK-1 has been identified as a new target in depression, and its antagonists might become effective antidepressants. In mice, deletion of the TREK-1 gene results in a depression-resistant phenotype that mimics antidepressant treatments. Here, we validate in mice the antidepressant effects of spadin, a secreted peptide derived from the propeptide generated by the maturation of the neurotensin receptor 3 (NTSR3/Sortilin) and acting through TREK-1 inhibition. NTSR3/Sortilin interacted with the TREK-1 channel, as shown by immunoprecipitation of TREK-1 and NTSR3/Sortilin from COS-7 cells and cortical neurons co-expressing both proteins. TREK-1 and NTSR3/Sortilin were colocalized in mouse cortical neurons. Spadin bound specifically to TREK-1 with an affinity of 10 nM. Electrophysiological studies showed that spadin efficiently blocked the TREK-1 activity in COS-7 cells, cultured hippocampal pyramidal neurons, and CA3 hippocampal neurons in brain slices. Spadin also induced in vivo an increase of the 5-HT neuron firing rate in the Dorsal Raphe Nucleus. In five behavioral tests predicting an antidepressant response, spadin-treated mice showed a resistance to depression as found in TREK-1 deficient mice. More importantly, an intravenous 4-d treatment with spadin not only induced a strong antidepressant effect but also enhanced hippocampal phosphorylation of CREB protein and neurogenesis, considered to be key markers of antidepressant action after chronic treatment with selective serotonin reuptake inhibitors. This work also shows the development of a reliable method for dosing the propeptide in serum of mice by using AlphaScreen technology. These findings point out spadin as a putative antidepressant of new generation with a rapid onset of action. Spadin can be regarded as the first natural antidepressant peptide identified. It corresponds to a new concept to address the treatment of depression.

## Abstract 2

We found that spadin, a natural peptide derived from sortilin, blocks the mouse TREK-1 channel and might be an efficient and fast-acting antidepressant.

## Author Summary

Depression is the most common of psychiatric illnesses, with prevalence estimates ranging from 5% to 20% within the general population. The design of effective treatments for this disorder is a challenging process, and the use of antidepressants has an overall low clinical efficacy as full remission only occurs in one-third of the patients. Moreover, the time between initial treatment and beneficial effects is relatively protracted. These limitations confirm the need to find new biological targets and drugs for the treatment of depression. We recently identified a conserved mouse potassium channel protein called TREK-1 (KCNK2) as a new target for treating depression. Here, we demonstrate that spadin, a natural peptide derived from a propeptide released in blood, is able to block the TREK-1 channel activity and has an antidepressant effect in mouse models of depression. We showed that spadin is an efficient antidepressant in mice that acts much faster (4 d versus several weeks) than fluoxetine, the most commonly used antidepressant. Our results with spadin in mice highlight the potential for novel and more efficacious treatments for depression in humans.

## Introduction

Recently, mouse models of depression have highlighted the putative role of the TREK-1 channel in the mechanisms of action of antidepressants. Deletion of the TREK-1 gene (also called *kcnk2*) results in a depression-resistant phenotype that mimics treatment with antidepressants [1]. TREK-1-deficient mice (*kcnk2* ^−/−^) display an increased efficiency of 5-HT neurotransmission, a blunted corticosterone response to stress and an increased neurogenesis induced by selective serotonin reuptake inhibitors (SSRIs). The involvement of the TREK-1 protein in mood regulation may be related to its two following specific properties: TREK-1 (1) is directly inhibited by SSRIs [1] and by activated protein kinases A and C and (2) is potentially linked to G-protein–coupled receptors like the 5-HT_1A_ receptor [2],[3], suggesting that this channel may participate in a 5-HT_1A_ receptor-dependent negative feedback loop. More interestingly, the Star*D study has identified an association between the existence of four genetic variants (SNPs) in the TREK-1 locus and resistance to multiple antidepressant classes [4]. All these findings indicate that (1) genetic variations in TREK-1 may identify individuals at risk for depression treatment resistance and (2) a search of selective blockers of TREK-1, hitherto not available, might potentially lead to a new generation of antidepressants.

Growing evidence indicates that trafficking and addressing as well as functional properties of native ion channels depend on their lipidic and proteic environments. K^+^ channels are known to interact with partner proteins that are crucial for their regulation. To date, the only identified partner proteins of TREK-1 channels are the A-kinase anchoring protein AKAP150 [5] and the microtubule-associated protein Mtap2 [6] that enhance TREK-1 channel surface expression and current densities. As a consequence of both its role in the sorting of membrane proteins and of a cerebral localization similar to that of TREK-1 [7],[8], we investigated the possible role of the neurotensin (NT) receptor 3 (NTSR3, also called gp95/sortilin) [9] in the regulation of the channel function. NTSR3/Sortilin is a 95–100-kDa type-1 membrane protein, consisting of a large luminal domain, a single transmembrane segment, and a short C-terminal cytoplasmic tail. A large part of NTSR3/Sortilin is localized at the level of the Golgi apparatus where the protein triggers intracellular functions of trafficking. Indeed, the C-terminus of NTSR3/Sortilin interacts with the VHS domain of the sorting protein GGA2 (Golgi-localizing, g-adaptin ear homology domain, ADP-ribosylation factor-binding protein) [10]. This interaction confers to NTSR3/Sortilin the property to sort SAP (sphingolipid activator proteins) to lyzosomes [11]. Depending on its cellular location, NTSR3/Sortilin may also act as a receptor or a co-receptor and binds NT, the precursor of the Nerve Growth Factor (proNGF), the receptor-associated protein (RAP), the lipoprotein lipase, and the propeptide released from its precursor form. For example, this receptor is essential to proNGF induction of neuronal cell death via a complex formed with the p75^NTR^ within the cell membrane [12]. In the rat brain, NTSR3/Sortilin as well as TREK-1 are highly expressed in cerebral structures involved in the pathophysiology of depression [13], such as prefrontal and cingulate cortice, amygdala, hippocampus, nucleus accumbens, dorsal raphe, and hypothalamus [7],[8]. NTSR3/Sortilin is synthesized as a proform (prosortilin) that, in late Golgi compartments, is converted to the functional ligand-binding receptor by cleavage and release of a 44 residue N-terminal propeptide (Gln^1^-Arg^44^, propeptide) by furin [14]. Propeptide binds to the mature receptor with a high affinity (Kd∼5 nM). Structure-function relationship studies have identified that the peptide Gln^1^-Arg^28^ was as efficient on the binding activity as the entire propeptide Gln^1^-Arg^44^, whereas the affinity of the peptide Gln^1^-Arg^16^ was very low [15]. Therefore, we designed the peptide spadin by conserving the sequence 17–28 in which we added the sequence 12–16 (APLRP) in order to maintain conformational stress. This partial propeptide (Ala^12^-Arg^28^) was tested for its potential effects on TREK-1 channel regulation and for its validation as an antidepressant drug in five behavioral models of depression.

## Results

### NTSR3/Sortilin and Spadin Interact with the TREK-1 Channel

**Figure 1:**
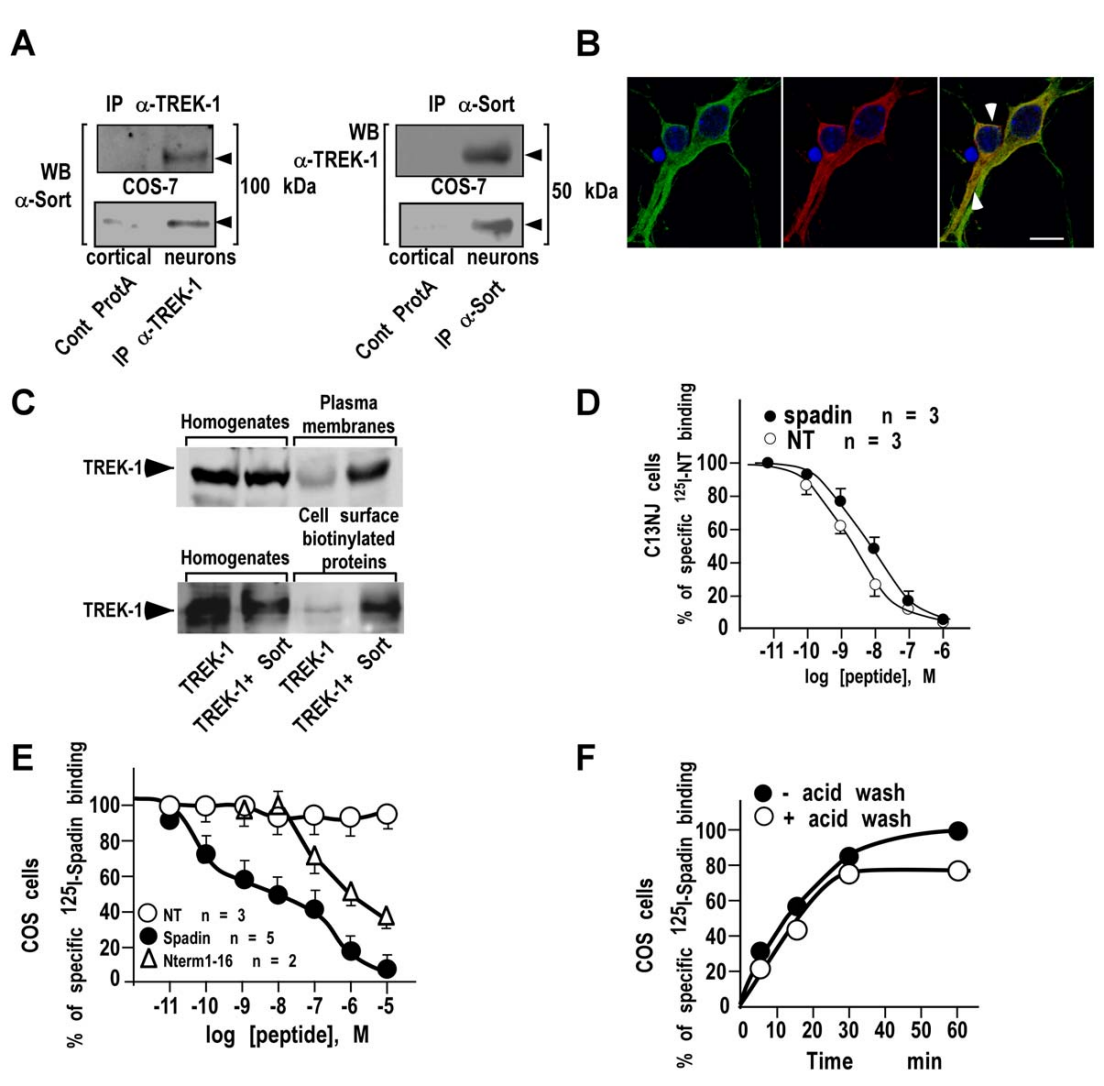
NTSR3/Sortilin and Spadin interact with the TREK-1 channel. (A) Immunoprecipitation of NTSR3/Sortilin with anti-TREK-1 antibodies (IP α-TREK-1) or of TREK-1 with anti-NTSR3/Sortilin antibodies (IP α-Sort) from transfected COS-7 cells or mouse cortical neurons. Immunoprecipitated proteins were subjected to Western blots and revealed using anti-sortilin (WB: α-Sort) or anti-TREK-1 (WB: α-TREK-1). (B) Double immunofluorescence labeling of TREK-1 (Green) and NTSR3/Sortilin (Red) in mouse cortical neurons. Nuclei were labeled using Dapi (Blue) and co-localized proteins were visualized using merge images (arrows); scale bar, 10 µm. (C) Influence of NTSR3/Sortilin on the expression of TREK-1 at the plasma membranes. COS-7 cells were transfected with TREK-1 in the absence or in the presence of NTSR3/Sortilin. Crude homogenates, purified plasma membrane proteins, or cell surface biotinylated proteins were subjected to Western blot analysis and revealed using anti-TREK-1 antibodies. (D) Competition between ^125^I-NT and unlabeled Spadin (closed circles) or NT (open circles) for binding to C13NJ cell homogenates. Each point represents the mean of duplicate determinations from 3 independent experiments. (E) Competition between ^125^I-Spadin and unlabeled Spadin (closed circles), NT (open circles) or N-terminal fragment Gln1-Arg 16 (Nterm1-16, open triangles) for binding to TREK-1 transfected COS-7 cell homogenates. Each point represents the mean of duplicate determinations from 2 to 5 independent experiments. Note that non-transfected COS-7 cells were totally devoid of ^125^I-Spadin binding. (F) Association kinetics of ^125^I-Spadin binding to COS-7 cells transfected with TREK-1. At the indicated times, cells were either washed twice with 500 µl of binding buffer (closed circles) or treated with 500 µl of acid-NaCl buffer for 2 min (open circles).

**Figure 2:**
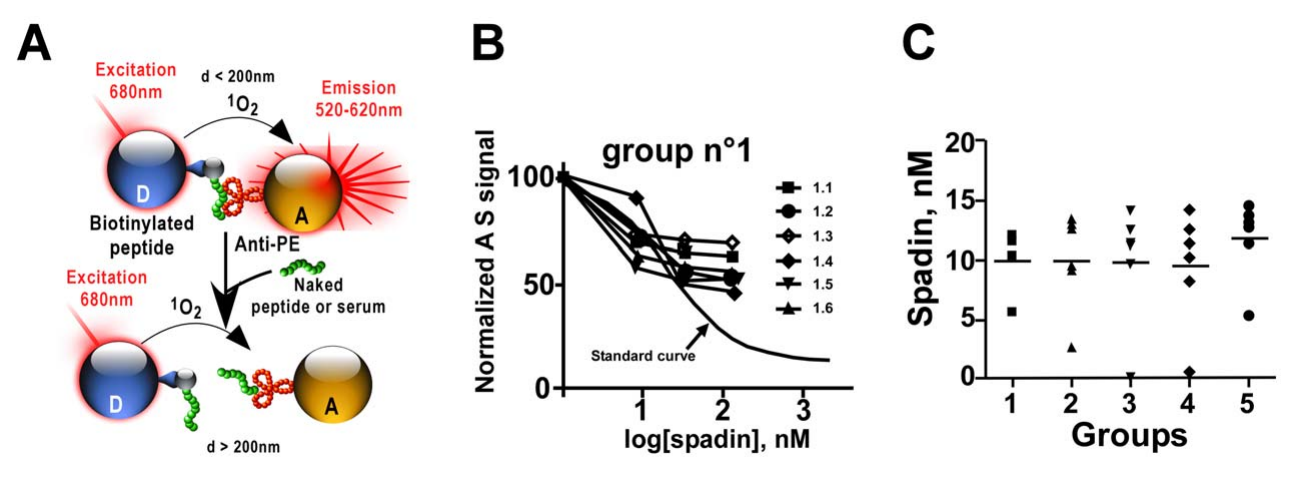
AlphaScreen assays. (A) Principles of AlphaScreen technology. Donor and acceptor microbeads can be coated with target-specific antibody, proteins, or secondary reagents (streptavidin, glutathione, nickel). A signal is produced when the AlphaScreen acceptor, A, and donor, D, beads are brought into proximity by a molecular interaction occurring between the binding partners captured on the beads. Laser excitation at 680 nm causes ambient oxygen to be converted to the singlet state by photosynthesizers on the donor bead. These react with chemiluminescent agents on the Acceptor bead only when the latter is in close proximity, emitting light at 520–620 nm. Here, we illustrate a competition protocol between seric propeptide, PE, and interacting donor beads, D, coupled-biotinylated spadin (b-spadin) with antibodies anti-propeptide (anti-PE) coupled on acceptor beads, A. (B) An example of competition curve obtained with one group (n° 1) of 6 mice (1.1 to 1.6) among 5 different groups (other curves are presented in the Figure S1). Values obtained are compared to the standard curve. (C) Seric concentrations of the full length propeptide calculated for the 5 groups from competition experiments as shown in (B).

In an attempt to detect a physical and functional interaction between the NT receptor and the potassium channel, we first performed an immunoprecipitation of TREK-1 and NTSR3/Sortilin. Experiments were performed on either mouse cortical neurons or COS-7 cells co-expressing both proteins. Each antibody immunoprecipitated the tested partner, *i.e.* NTSR3/Sortilin [8] precipitated with the TREK-1 antiserum (Figure 1A left panel) [16] and TREK-1 with the anti-NTSR3/Sortilin antibody (Figure 1A right panel), in both COS-7 cells and cortical neurons. We also demonstrated that both endogenous proteins were colocalized in mouse cortical neurons (Figure 1B). Then, we investigated the influence of NTSR3/Sortilin expression on the sorting of TREK-1 to the plasma membranes. The expression of TREK-1 within the plasma membranes, measured either by preparing purified plasma membranes or by using cell surface biotinylation, was enhanced (by a factor 3 and 6, respectively) when COS-7 cells were cotransfected with NTSR3/Sortilin (Figure 1C), confirming the interaction between the two proteins, at least during the channel sorting. This interaction between TREK-1 and NTSR3/Sortilin led us to examine whether NT and/or the partial NTSR3/Sortilin propeptide (i.e. spadin) were able to act on TREK-1 channel activity. We first characterized the affinity of spadin on C13NJ, a microglial cell line expressing only NTSR3/Sortilin as a receptor for NT, and devoid of TREK-1 (unpublished data). Similarly to NT, spadin bound to NTSR3/Sortilin by displacing the binding of ^125^I-NT with an affinity of 8 nM, identical to that previously found with the full length propeptide (Figure 1D) [17]. Since NT plays a role on C13NJ migration in a wound-healing assay and that the full length propeptide antagonizes this effect [17], we tested in the same assay the spadin effect on NT-induced cell migration. In serum free medium containing 10 nM NT, the number of cells that migrated corresponded to 35.1%±2.3% of the number of migrating cells in the presence of 10% fetal calf serum (FCS). In absence of stimulation, only 4%±1% of cells migrated. The 10 nM NT-induced cell migration was totally abolished in the presence of 1 µM spadin and remained to the basal level (6.2%±1.3%) (Figure S1). This result confirms that spadin displays identical binding and functional properties as those of the full length propeptide. Then, we performed competition experiments between ^125^I-labelled spadin and unlabelled spadin, NT or the N-terminal fragment of the full length propeptide Gln1-Arg16 on membrane homogenates from COS-7 cells transfected or not with TREK-1. Figure 1E shows that spadin bound specifically to TREK-1 with an affinity of about 10 nM. This binding was selective since NT was unable to displace the binding of ^125^I-spadin and the N-terminal fragment Gln1-Arg16 bound to TREK-1 with a very low affinity (1 µM) close to that reported with NTSR3/sortilin (Figure 1E) [15]. The weak effect of the N-terminal fragment Gln1-Arg16 was confirmed by electrophysiological recordings on TREK-1 transfected COS-7 cells (Figure S2). We also performed association kinetics of ^125^I-labelled spadin on whole COS-7 cells expressing TREK-1 at 37°C. The radioactivity associated with cells reached a plateau within 30 min. Removal of surface-bound radioactivity by acid-NaCl wash revealed that about 80% of total ^125^I-labelled spadin bound at this time was intracellular, indicating that spadin was internalized with TREK-1 following interaction (Figure 1F). These data strongly suggest that NTSR3/Sortilin constitutes a sorting partner of the TREK-1 channel. We hypothesized that when both proteins reach the plasma membrane, the propeptide, which is cleaved in the Golgi apparatus, can be released. Then, it may bind to NTSR3/Sortilin and/or to TREK-1 for tuning the channel activity, by blocking a part of the expressed channels, and by promoting their internalization. However, for such a mechanism to be functionally effective under in vivo physiological conditions, the propeptide has to be released into the blood circulation. To validate this possibility, we therefore developed the Alpha Screen (Amplified Luminescent Proximity Homogenous Assay) technology for dosing the propeptide or spadin in serum samples from mice [18],[19],[20]. This method is a bead-based non-radioactive and homogenous proximity assay used to measure the interaction between biological binding partners (Figure 2A–B). The principle of this technology relies on the use of a Donor bead and an Acceptor bead that generate a light signal when brought into proximity (<200 nm). Upon laser excitation at 680 nm, the Donor beads, containing a photosensitizer, will generate short-lived singlet oxygen that can diffuse only a short distance before returning to the ground state. The Acceptor beads, containing chemiluminescers and fluorophores, will emit an amplified light signal measurable at 600 nm (Figure 2A). Using this approach, we calculated seric propeptide concentrations of 5 groups of 6 littermate mice. Interestingly, the mean concentration values of the 5 groups were very close to each other, with a value of about 10 nM (Figures 2C and S3). These data clearly indicated that the propeptide is released into the blood circulation. On this basis, we next investigated its effects, and by extension those of spadin as well, on TREK-1 channel activity.

### Effects of Spadin on the TREK-1 Channel Activity

**Figure 3:**
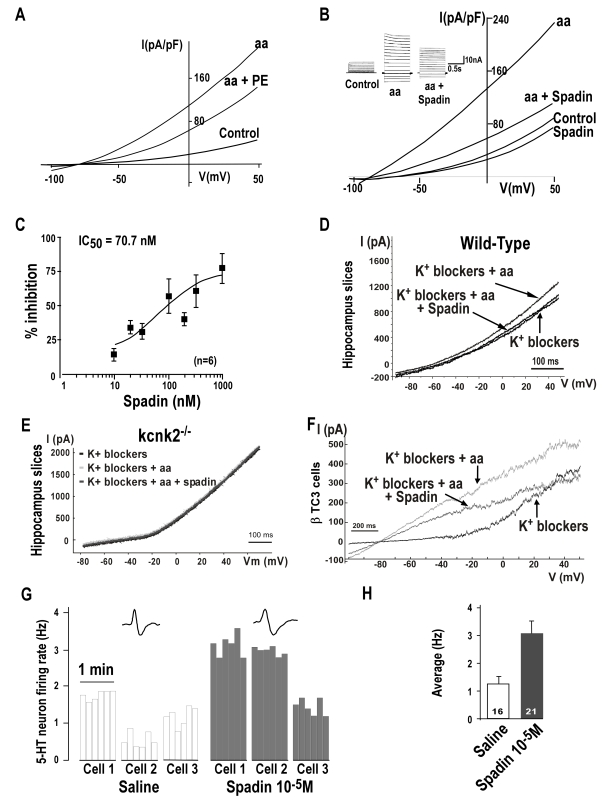
Effects of Spadin on the TREK-1 channel activity. (A–B) Whole-cell currents measured in COS-7 transfected cells in presence of potassium blockers (K^+^ blockers, 10 mM tetraethyl ammonium (TEA), 3 mM 4-aminopyridine (4-AP), 50 nM charybdotoxin, 10 µM glibenclamide, 100 nM apamin). Cells were clamped at −80 mV and voltage changes were either applied by ramp from −100 to 50 mV, 1 s in duration (A, B main panel) or by 10mV steps from −100 to 40 mV, 1.5 s in duration (B inset). Currents were recorded after TREK-1 activation by 10 µM arachidonic acid (aa) and aa + propeptide (PE, 500 nM) (A) or aa + Spadin (100 nM) (B). Native currents were recorded in absence (Control) and in presence of spadin (Spadin 100 nM, B). Peptides were applied *via* the bath medium. (C) Dose-dependent spadin inhibition of TREK-1 currents, IC_50_ value at 0 mV is of 70.7 nM. Currents were measured in presence of 10 µM aa. (D–E) Native currents recorded in the presence of K^+^ blockers after stimulation by 10 µM of aa on CA3 pyramidal neurons from hippocampus slices in wild-type mice (D) or in *kcnk2* deficient mice (*kcnk2* ^−/−^) (E) in the presence or the absence of spadin (1 µM). Currents were elicited by a ramp from −100 mV to 50 mV. (F) Native currents on β-TC3 cell line in similar experimental conditions as (D–E). (G–H) Effect of spadin on the firing rate of DRN 5-HT neurons. Spadin (10^−5^ M in a 100 µl bolus) or its vehicle was i.p. administered. Recordings started 30 min after the injection and were performed for a maximal duration of 210 min thereafter. (G) Main panel: Samples of “descents” performed along the DRN, showing typical integrated firing rate histograms in a vehicle- (left panel) or in a spadin-treated (right panel) animal. Each cluster represents the electrical activity of one neuron, each bar representing the average number of recorded action potentials per 10 s. Insets, examples of action potential waveforms of 5-HT neurons. (H) 5-HT neuron firing activity, calculated on the basis of all the cells recorded within the successive tracks performed along the DRN. Values at the bottom of each column indicate the total number of neurons recorded (n = 4 mice in both groups).

As previously described [21], TREK-1 basal channel activity was strongly and reversibly activated by arachidonic acid (aa, 10 µM), which induced a typical TREK-1 background current, characterized by outward rectification reversed at the predicted value for E_K+_. Using the whole-cell patch-clamp technique on TREK-1 transfected COS cells, we first assessed the ability of the full length peptide to inhibit the TREK-1 channel activity. COS-7 cells were chosen because they weakly express the NTSR3/Sortilin receptor (unpublished data). Indeed, 500 nM of propeptide was able to block 41%±5% (n = 4) of the aa stimulated TREK-1 current measured at 0 mV (Figure 3A). Then, we tested spadin in the same experimental conditions. As expected [15], we found that spadin displayed a better affinity than the propeptide, since 100 nM of spadin were able to block 63%±12% (n = 16) of the TREK-1 current stimulated by aa (Figure 3B). A spadin dose-response experiment indicated an IC_50_ value of 70.7 nM at 0 mV (Figure 3C).

In order to confirm the action of spadin on TREK-1, by using brain slices we directly recorded hippocampal CA3 pyramidal cells, a cellular network that endogenously expresses both TREK-1 and NTSR3/Sortilin [7],[8]. Figure 3D depicts the currents obtained following a ramp of potential in a CA3 neuron in the presence of a cocktail of K+ blockers that have no effect on TREK-1 [21]. In 12 out of 28 recorded neurons, arachidonic acid increased the amplitude of the remaining current by 23.3%±4.8% (n = 12). Spadin blocked 90.8%±6.0% (n = 12) of this effect. Interestingly, the peptide alone inhibited 14.9%±5.6% (n = 8) of the current recorded in the presence of potassium blockers (unpublished data), as did fluoxetine (13.0%±3.8%, n = 5) (unpublished data). Even if an effect of spadin on cationic channels cannot be totally excluded, the inhibitory effect of spadin on arachidonic acid-induced current in CA3 neurons from wild-type mice was totally absent in the same experimental conditions in the *kcnk2* ^−/−^ mice (Figure 3E). This result clearly demonstrates that the current blocked by spadin is supported by TREK-1 channel. The inhibitory effects of spadin on endogenous TREK-1 were also measured in cultured pyramidal neurons from hippocampus (49.7% inhibition with 1 µM of spadin, Figure S4) and in the non-neuronal βTC3 pancreatic cell line (36% inhibition with 1 µM of spadin, Figure 3F) that endogenously express both proteins (Mazella, personal communication).

### Effect of Spadin on the Dorsal Raphe Nucleus (DRN) 5-HT Neurotransmission

Since we have previously demonstrated that the deletion of the TREK-1 gene results in an increase of the 5-HT neuron firing rate in the DRN [1], we tested the effect of spadin on the same neurons. We performed unitary extracellular recordings of these 5-HT neurons in anesthetized animals (see Text S1). We constituted two groups of mice, which received via an i.p. injection either spadin (10^−5^ M in a 100 µl bolus) or its vehicle (distilled water). Starting 30 min after the injection, 3 to 4 successive descents were performed along the DRN, for a total of 4–8 cells recorded per animal (examples are given in Figure 3G). For each neuron, the discharge was monitored during 60 s. In vehicle-injected mice, we found a value of 1.26±0.27 Hz, whereas after administration of spadin, the mean firing rate of DRN 5-HT neurons was significantly elevated up to 3.1±0.7 Hz (Figure 3H) (one-way ANOVA, F(_1,36_) = 4.4, *p*<0.05), corresponding to a +146% increase. As shown in Figure 3G (right panel), several neurons found in spadin-injected mice discharged at up to 3, 5, or even 6 Hz, whereas most of the frequencies found in the saline group were in a normal range (1–1.7 Hz) (Figure 3G left panel). The average 5-HT neuron firing activity in spadin-treated animals was almost identical to that observed in *kcnk2* ^−/−^ mice (Figure 3H) [1]. Very similar results were obtained when spadin was i.p. injected in rats (Figure S5A–B).

Taken together, these results indicate that TREK-1 and NTSR3/Sortilin are not only associated within the plasma membrane but that spadin interacts directly with TREK-1 to functionally inhibit its activity. These results prompted us to test thereafter the antidepressant-like effects of spadin in behavioral, morphological, and molecular models.

### Acute, Subchronic, and Chronic Spadin Treatments Induce Antidepressant Effects

**Figure 4:**
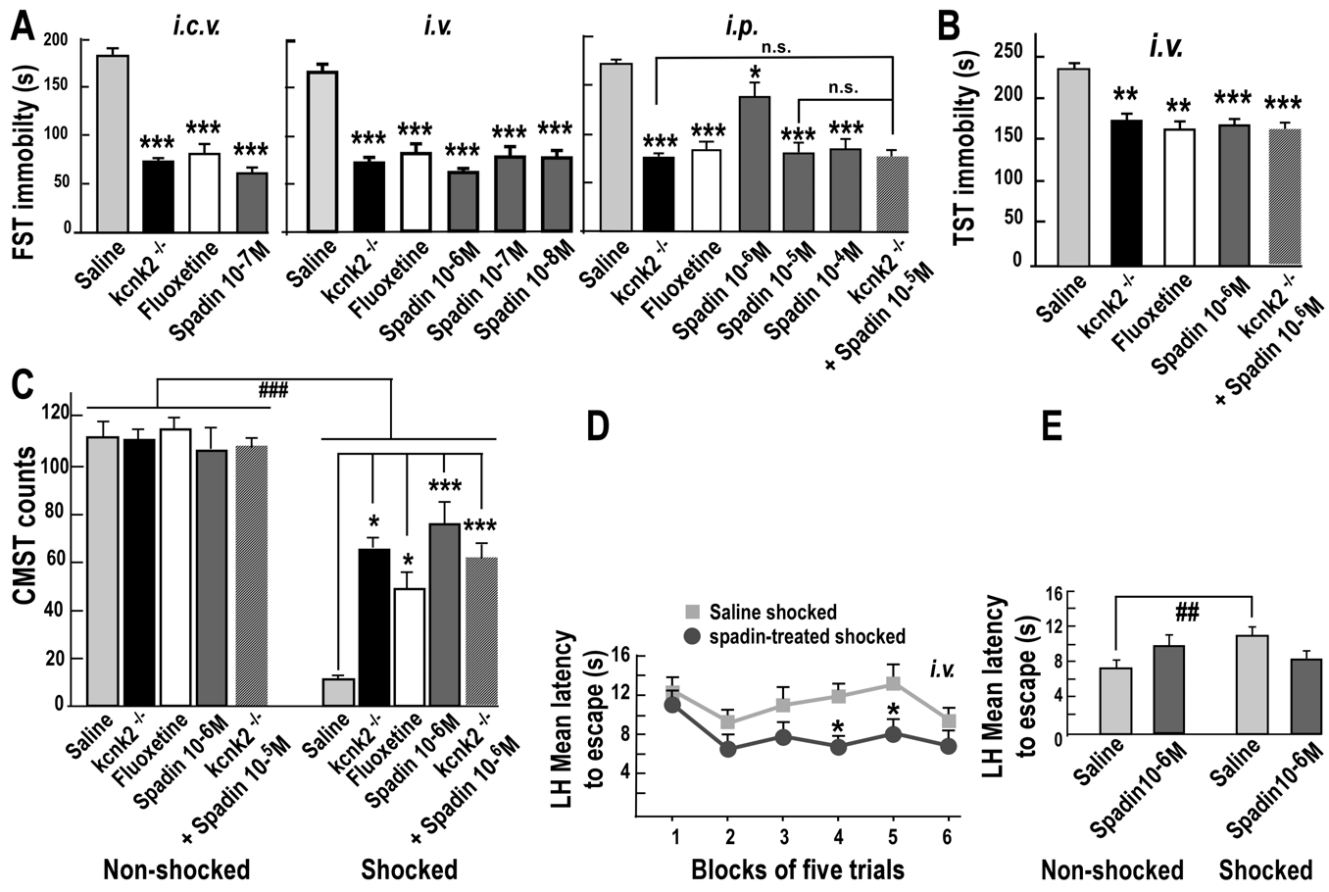
Acute antidepressant effects of Spadin. (A–E) Acute treatments: Spadin (10^−4^ to 10^−8^ M) or Fluoxetine (3 mg/kg) or Saline solutions were injected 30 min before the test in wild-type and *kcnk2* ^−/−^ mice (A, B, C). (A) Forced Swimming Test (FST, n = 10 per group), spadin-treated mice had a shorter time of immobility comparable to those obtained with *kcnk2* ^−/−^ or fluoxetine-treated mice, whatever the way of spadin administration: intracerebroventricular (i.c.v., *n* = 14 per group) (one-way ANOVA, *F*_3,55_ = 79.53, ***p<0.001 versus saline-treated mice), intravenous (i.v., n = 8 per group except for fluoxetine and *kcnk2* ^−/−^ groups, n = 6) (one-way ANOVA, *F*_5,43_ = 26.27, ***p<0.001 versus saline-treated mice) or intraperitoneal (i.p., n = 10 per group except for *kcnk2* ^−/−^, n = 5 ) (one-way ANOVA, *F*_3,34_ = 40.58, *p<0.05, ***p<0.001 versus saline-treated mice). (B) Tail Suspension Test (TST, n = 15 for saline and spadin groups, and n = 9 for fluoxetine and *kcnk2* ^−/−^ groups), i.v. spadin-treated mice had a shorter immobility score comparable to those obtained with *kcnk2* ^−/−^ or fluoxetine-treated mice (one-way ANOVA, *F*_3,47_ = 11.40, **p<0.01, ***p<0.001 versus saline-treated mice). (C) Conditioned Motility Suppression Test (CMST, n = 10 per group). Two-way ANOVA showed significant effects of shocks (*F*_1,62_ = 254.1, *p*<0.001), treatment (*F*_3,62_ = 3.87, *p*<0.01) and an interaction between these two factors (*F*_3,62_ = 8.83, *p*<0.001). ^###^ *p*<0.01 versus non-shocked mice. In the shocked groups, spadin treatment reversed the freezing state induced by the shock training in saline-treated mice (78±7 versus 14±2 counts, respectively). This effect was stronger than those observed for *kcnk2* ^−/−^ or fluoxetine-treated mice (one-way ANOVA, *F*_3,39_ = 10,87, *p<0.05, ***p<0.001 versus saline-treated mice). Counts are the number of squares crossed plus the number of climbings. (D and E) Learned Helplessness test (LH, n = 12 per group). Shocked spadin-treated mice showed shorter escape latencies than saline-treated mice. Two-way ANOVA showed significant effect for treatment (F_1,110_ = 7.93, *p* = 0.01) and for assay (F_5,110_ = 3.56, *p* = 0.005 , *p<0.05 in shocked groups). (D) Mean escape latencies ± SEM averaged in 6 blocks of 5 trials, and (E) mean overall latency ± SEM to escape across trials 1–30 as a function of spadin treatment. Two-way ANOVA (Shocks×Treatment) showed an interaction between these two factors (F_1,44_ = 6.9, *p* = 0.012). ^##^ *p* = 0.007 for non-shocked saline-treated mice versus shocked saline-treated mice.

We used five behavioral tests predicting an antidepressant response (FST, TST, CMST, LH, and NSF) (see Text S1) similar to these used in our previous work on the depression-resistant phenotype of TREK-1 deficient mice [1]. Spadin efficacy was first assessed in the Forced Swimming Test (FST) [22], which is a highly reliable predictor for antidepressant potential [13]. Spadin was administered 30 min before the test by intracerebroventricular (i.c.v.), intravenous (i.v.), or intraperitoneal (i.p.) route at doses of 10^−4^ to 10^−8^ M. Its effects were compared to the behavior observed in *kcnk2* ^−/−^ mice and in wild-type mice treated with the efficient SSRI fluoxetine (i.p., 3 mg/kg). When placed in an inescapable cylinder of water, spadin-treated mice exhibited reduced floating or immobility times in the three modes of injection with respect to their saline-treated counterparts (Figure 4A). The immobility is interpreted as “a state of despair,” in that the animal is believed to have lost its motivation for escape-oriented behaviors. The dose-responses of spadin showed that the highest reduced immobility times (*p*<0.001) were observed at the dose of 10^−7^ M in i.c.v. (66.8%), 10^−6^ M in *i.v.* (62.9%), and 10^−5^ M in i.p. (55.30%) administration. The magnitude of the antidepressant behavior was similar to that observed in fluoxetine-treated wild-type and saline-injected *kcnk2* ^−/−^ mice. Then, we determined the effect of an acute i.v. spadin administration (10^−6^ M) in the Tail Suspension Test (TST, Figure 4B), which is often used to predict antidepressant efficacy [23],[24], and in the test of Conditioned Suppression of Motility (CMST, Figure 4C), sensitive to antidepressants but not to anxiolytic drugs [25]. In the TST, injection of spadin in wild-type mice 30 min before the test significantly reduced immobility times when compared to saline-treated wild-type mice (*p*<0.001). The antidepressant effect was not statistically different to that observed in fluoxetine-injected mice or *kcnk2* ^−/−^ mutants (*p*>0.05; Figure 4B). In the CMST, shocked mice treated with saline displayed a marked suppression of motility (CS, conditioned suppression; 9.1% of the saline non-shocked group) when they were returned to the cage in which they had previously received electric shocks (Figure 4C). Similarly to what was observed in saline-treated *kcnk2* ^−/−^ mice, the administration of spadin (i.v., 10^−6^ M) significantly reduced (by 84.4%) the CS of motility without increasing motility in the corresponding non-shocked group (Figure 4C). In these three tests (FST, TST, and CMST), the injection of spadin in *kcnk2* ^−/−^ mice (see Text S1) did not induce any change (Figure 4A–C), indicating that there was no additional effects of spadin in the absence of the TREK-1 channel. The subsequent experiments were therefore performed only in wild-type mice. We subjected mice to the Learned Helplessness test (LH, Figure 4D–E), validated as a sensitive model of depression [13],[26]. Compared with non-shocked mice (i.e. that had not been exposed to inescapable shocks), learned helpless (shocked) mice treated with saline showed a significant increase of escape latencies, when tested for their escape performance abilities 1 d after exposure to inescapable shocks (Figure 4D–E). However, an acute spadin treatment (i.v., 10^−6^ M) provoked significant reduced escape latencies after training (25.4%) compared to saline-treated mice, demonstrating a strong antidepressant effect. Because changes in the motor activity induced by the different drugs used could influence the results, the motor behavior was measured following i.p. treatments. Neither spadin nor fluoxetine had any effect on mouse locomotion analyzed in short- or long-time after the drug injection (Figure S6).

**Figure 5:**
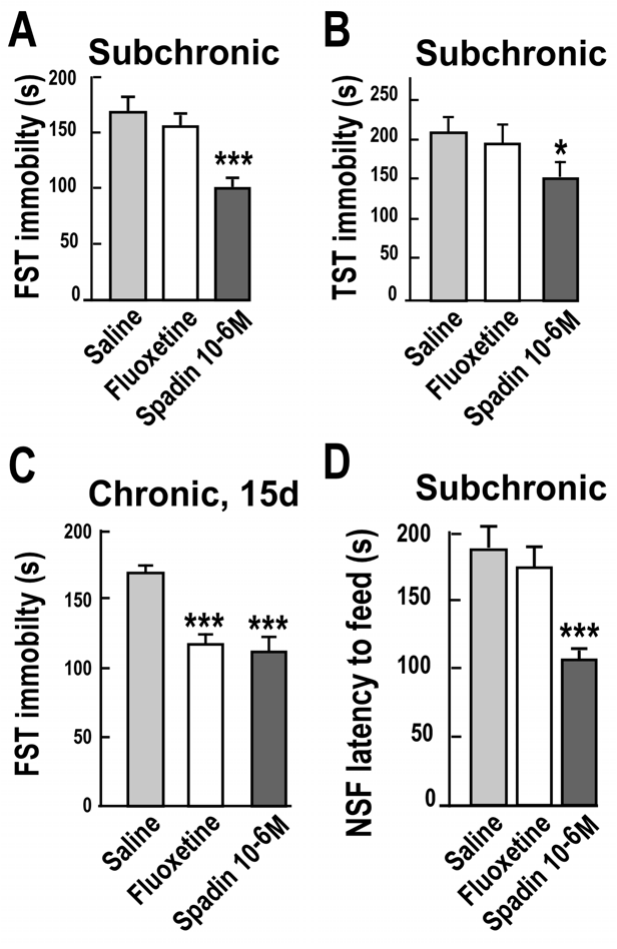
Subchronic and chronic antidepressant effects of Spadin. Subchronic treatments: Spadin (10^−6^ M), Fluoxetine (3 mg/kg), or Saline solutions were i.v. injected in a 100 µL bolus once a day for 4 successive d before the test. In chronic treatments, spadin (10^−6^ M) and fluoxetine (1 mg/kg) were i.v. injected in a 100 µL bolus once a day for 15 successive d. For each test there were 8 animals per group. (A) In FST (one-way ANOVA, *F*_2,23_ = 26.08, ***p<0.001) and (B) TST (one-way ANOVA, *F*_2,24_ = 9.8, *p<0.05 versus saline-treated mice), spadin induced similar behaviors than those obtained with the acute treatment, whereas fluoxetine was without effect [30]. (C) In FST, chronic treatment with spadin or fluoxetine significantly reduced the time of immobility (one-way ANOVA, F_2,26_ = 25.08, ***p<0.001 versus saline-treated mice). (D) NSF paradigm: at the end of the 4 d treatment, animals were food deprived for 1 d and then measured for their latency to feed. Spadin treatment significantly reduced the latency to feed when compared to saline or fluoxetine treatments (*t* test, ***p<0.001 versus saline-treated mice). In all graphs, data are expressed as means ± SEM.

Current antidepressants are clinically effective only after several weeks of administration. They increase the efficacy of 5-HT transmission at the postsynaptic levels [27],[28], but the initial elevation of 5-HT also induces the stimulation of inhibitory 5-HT_1A_ autoreceptors within the DRN, counteracting the facilitation of 5-HT transmission related to terminal reuptake blockade [28]. The existence of this presynaptic effect is believed to be responsible for the 2 to 6 wk delay before the onset of the antidepressant's therapeutic action, as this period corresponds to the time required for 5-HT_1A_ autoreceptors to desensitize [28]. Based on these observations, it has been proposed that a direct facilitation of 5-HT firing rate in the DRN should be a requirement for a faster onset of antidepressant action [29]. Interestingly, we observed an increase in the firing activity of DRN 5-HT neurons (Figure 3G). Obviously, such results raise the possibility that spadin could exert a rapid onset of action. Hence, we tested the potential antidepressant effect of spadin administered during 4 d (subchronic treatment) using both the FST and TST tests. As shown in Figure 5A–B, a 4-d treatment with spadin (i.v., 10^−6^M) significantly reduced the time spent immobile by 43.2% in FST (*p*<0.01) (Figure 5A) and by 28.1% in TST (Figure 5B). In contrast, as previously observed [30], subchronic fluoxetine treatment had no effect when compared with saline (*p*>0.05). Both spadin and fluoxetine administered during 15 d (chronic treatment) significantly reduced the time of immobility to a similar extend (around 30%) in the FST paradigm (Figure 5C). This result shows that the antidepressant effect of spadin reached a maximal level after 4 d and maintained the same potency following long-term administration (15 d). The Novelty Suppressed Feeding test (NSF) is usually carried out for demonstrating antidepressant efficacy after chronic but not acute treatment [31]. Mice treated with spadin (i.v., 10^−6^ M) for 4 d showed a significant decrease in latency to feed relative to saline-injected animals (Figure 5D). As previously described [30], a 4-d regimen with fluoxetine (*i.p.*, 3 mg/kg) had no effect in the same condition (Figure 5D). None of the drugs tested produced a significant change in food consumption when mice returned in their home cage immediately after the test (unpublished data).

To verify that the antidepressant effects of spadin were not species-specific, we also tested its efficiency in rats, by using the FST test. An acute spadin injection (i.p., 10^−5^ M) 30 min before the test significantly reduced immobility time when compared with saline-treated rats (*p*<0.001). The antidepressant effect was not statistically different to that obtained with fluoxetine (20 mg/kg)-injected mice or *kcnk2* ^−/−^ mice (*p*>0.05; Figure S5C).

### Acute Spadin Treatment Does Not Affect Anxiety-Related Behaviors

**Figure 6:**
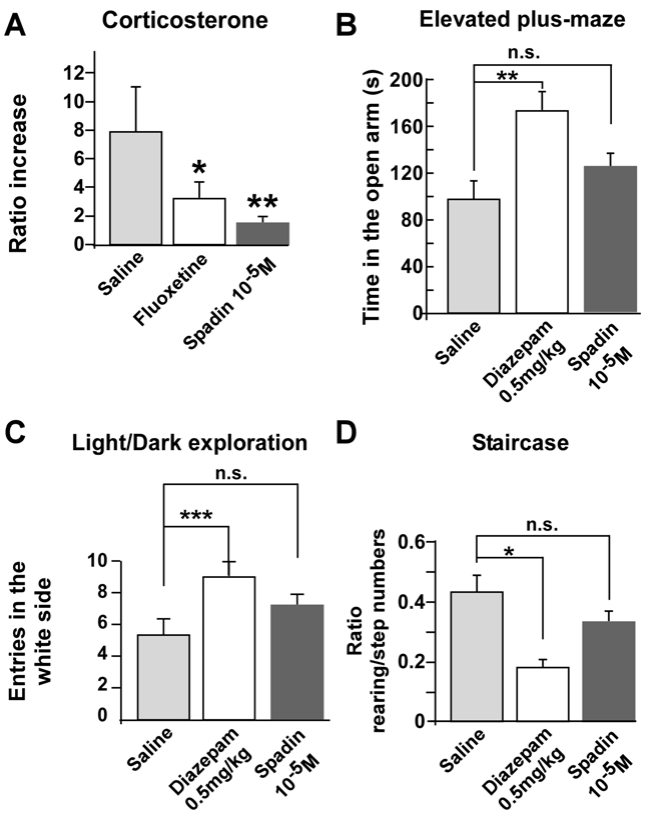
Effect of Spadin on stress and anxiety behaviors. (A) Decreased stress-induced serum levels of corticosterone in mice treated with spadin. We compared serum corticosterone concentrations (ng/ml) sampled in the morning in mice acutely treated with spadin (i.v, 10^−6^ M), saline or fluoxetine (i.p., 3 mg/kg) 30 min after a 10 min tube restraint (n = 10 per group). Data are expressed as increase of the ratio corticosterone levels 30 min after stress over basal corticosterone levels 30 min before restraint (one-way ANOVA, F_2,27_ = 18.30, *p<0.05, **p<0.01 versus saline-treated mice). (B) Effect of spadin (i.p, 10^−5^ M) and diazepam (i.p., 0.5 mg/kg) on time spent in the open arms (s) of the elevated plus-maze (n = 10 per group, one-way ANOVA, F_2,27_ = 8.75, **p<0.001 versus saline-treated mice). (C) Effect of spadin (i.p., 10^−5^ M) and diazepam (i.p., 0.5 mg/kg) on the total number of entries in the aversive white side in the light/dark transition test (n = 10 per group, one-way ANOVA, F_2,53_ = 7.65, ***p = 0.001 versus saline-treated mice). (D) Influence of spadin (i.p., 10^−5^ M) and diazepam (i.p., 0.5 mg/kg) on mouse performance in the staircase test. Data are presented as the ratio of number of rearings over the number of ascended steps (n = 10 per group, one-way ANOVA, F_2,44_ = 4.86, *p<0.05 versus saline-treated mice). In the three tests, mice were injected with either spadin or diazepam 30 min before the test. In all graphs, bars indicate SEM.

Stress and anxiety disorders lead to profound suffering and disability, which contributes to the development of depression in humans and plays a role in the severity and the recurrence of the disease [32]. The connection between stress, anxiety, and depression is often associated with elevated cortisol levels in depressed patients [33]. We have previously demonstrated that the deletion of the TREK-1 gene results in a hypoactivity of the hypothalamic-pituitary-adrenal (HPA) axis, known to be involved in the control of stress [1]. Here, we tested whether spadin (i.p. 10^−5^ M) reduced corticosterone levels 30 min after a 10 min tube restraint, a paradigm known to activate the HPA axis [34]. Figure 6A shows that the increase in corticosterone levels induced by stress were reduced by 79.5% and 59.1% in spadin- and fluoxetine-treated mice with respect to saline-treated animals.

Thereafter, to study whether spadin affects anxiety-related behaviors, we investigated its anxiolytic profile in the three mouse models of anxiety (elevated plus-maze, light-dark exploration, staircase) (see Text S1) that are the most commonly used [35]. Their most important feature resides in the predictive validity to detect anxiolytic potential. Avoidance behaviors are reduced by treatments with clinically efficacious anxiolytics, mainly by the benzodiazepine agonist class, including diazepam [36]. In the elevated plus-maze test, compared to diazepam (i.p., 0.5 mg/kg), which significantly reduced the time spent into the aversive open arms of the test apparatus (*p<0.05), spadin (i.p., 10^−5^M) had no effect (*p*>0.05) versus saline-treated mice (Figure 6B). Similarly, in the light/dark exploration test, spadin-treated mice did not make more transitions from the dark to the light compartment than did mice treated with saline (*p*>0.05). In contrast, diazepam significantly induced an increase in the number of light/dark transitions (*p<0.05) (Figure 6C). The staircase paradigm combines step-climbing, which serves as an index of exploratory and locomotor activity, and rearing, which serves as an indicator of anxiety. Exposure to diazepam induced a significant reduction in both rearing and step ascending behaviors, leading to a decrease of the rearing/step numbers ratio (**p<0.01, Figure 6D). In contrast, spadin had no effect as compared to saline-treated mice (*p*>0.05). Together, these results demonstrate that spadin has no anxiolytic activity, when compared with the well-known diazepam.

Since spadin exerted efficient effects after i.p. or i.v. administration, we evaluated its ability to pass through the blood-brain barrier. ^125^I-labelled spadin (1 nmol of spadin plus 2×10^6^ cpm ^125^I-spadin) was i.v. injected and animals were sacrificed 30 min following injection. The brains were rapidly removed and homogenized. The radioactivity recovered in the brain was acid-extracted, quantified, and analyzed by HPLC. These experiments indicated that 1/1,000 of spadin was recovered in the brain after i.v. injection (unpublished data). Identical amounts of spadin were recovered in the brain after i.p. injection under the same experimental conditions. The concentration of spadin recovered into the brain was estimated to stand around 10 nM, a concentration that corresponds to the affinity of spadin for TREK-1 and sufficient to be active on TREK-1 channel activity. This value was also in the same range of the IC_50_ determined by electrophysiological measurements (Figure 3C).

### Effects of a 4-d Treatment with Spadin on CREB-Phosphorylation and Hippocampal Neurogenesis

**Figure 7:**
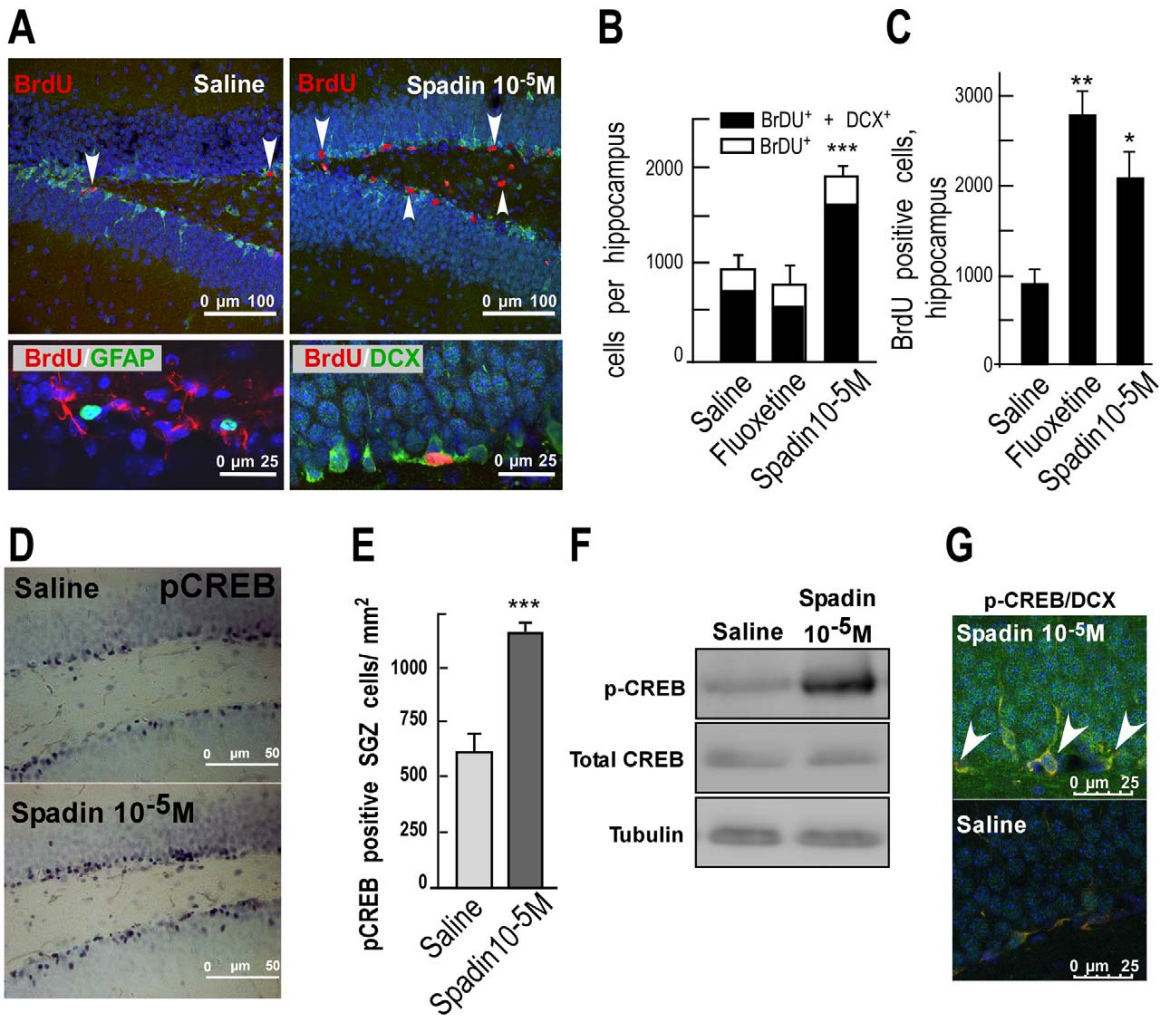
Effects of Spadin on neurogenesis and CREB activation. (A–B) Spadin increased neurogenesis (A). Top, representative photomicrographs of BrdU-labeled neurons in the dentate gyrus of the mouse hippocampus treated either with saline or with spadin (i.v., 10^−6^ M) for 4 d. Bottom, double labeling of BrdU-labeled neurons either with GFAP (glial marker) or with DCX (neuronal precursor marker), showing a co-localization only with DCX, and not with GFAP. (B) Quantitation of BrdU positive cells of hippocampus treated with saline, fluoxetine, or spadin (10^−5^ M) for 4 d. 85% of BrdU-labeled cells were positive to DCX. Data are number of BrdU^+^ or DCX^+^ cells in mouse hippocampus (*n* = 5) (F_2,53_ = 35.27; ***p<0.001 versus saline). (C) Quantitation of BrdU positive cells of hippocampus treated with saline, fluoxetine, or spadin (10^−5^ M) for 15 d (*n* = 5) (F_2,53_ = 19.43; *p<0.05, **p<0.01 versus saline). (D–G) Enhanced spadin treatment-induced CREB activation in the hippocampus, as assessed by measuring phosphoCREB (pCREB) immunoreactivity. (D) Immunological distribution of pCREB in the mouse hippocampus after a 4 d i.v. treatment. pCREB is phosphorylated in the cells near the subgranular zone (SGZ). (E) Quantification of pCREB positive cells/mm^2^ in hippocampal SGZ (n = 5) (*t* test; ***p<0.001). (F) Western blot analysis of pCREB level in hippocampus treated with saline or spadin (10^−5^ M). (G) Double immunofluorescent staining (examples are indicated by arrows) for pCREB and DCX positive hippocampal neurons treated with saline or spadin (10^−5^ M).

SSRIs and tricyclics are known to enhance neurogenesis in the subgranular zone (SGZ) of the dentate gyrus, but only after 2 or 3 wk of a chronic treatment [31],[37]. The concomitant increases of both the transcription factor cAMP response element-binding protein (CREB) and hippocampal neurogenesis in response to chronic antidepressant treatment, but not to non-antidepressant psychotic drugs, strongly suggest that CREB regulates hippocampal neurogenesis [13],[38]. We tested therefore whether spadin was able to induce an increase in hippocampal neurogenesis and a faster activation of CREB. We analyzed the neurogenesis in the dentate gyrus of the mouse hippocampus, by counting the number of progenitor cells that incorporate the DNA synthesis marker 5-bromo-2′deoxyuridine (BrdU) and that are differentiated into mature neurons [31],[37]. Interestingly, in the SGZ, a 4-d treatment with spadin (i.p. 10^−5^M) significantly increased by 2-fold the number of BrdU-positive cells with respect to saline conditions (Figure 7A–B). The neurogenic effect of spadin was maintained after a long-term administration (15 d, Figure 7C). In contrast, a 4-d regimen with fluoxetine had no effect on neurogenesis, but fluoxetine induced a significant increase in the number of BrdU-positive cells when it was administered during 15 d (Figure 7C). Dual labeling of BrdU and doublecortin (DCX), a specific marker of neuronal precursors [39], revealed that 85.2% of BrdU-labeled cells expressed DCX (Figure 7A right bottom panel, 7B). No colocalization of BrdU-positive cells with the astroglial marker GFAP (glial fibrillary acidic protein) was observed (Figure 7A left bottom panel).

The next step was to determine whether the enhanced adult spadin-induced neurogenesis was related to an increased hippocampal activation of CREB, as measured by its phosphorylation into pCREB. Compared with saline-treated mice, a 4-d treatment with spadin (i.p., 10^−5^M) induced a large increase of pCREB labeling, restricted to the specific SGZ region of mouse hippocampal tissue sections (Figure 7D). The counting of pCREB^+^ cells revealed that the spadin administration during 4 d significantly led to a 2-fold increase in the number of pCREB-labeled neurons when compared with the saline group (*p*<0.001, Figure 7E). Western blot analysis, which showed an immunoreactive band at 46 kDA corresponding to the phosphorylated active form of CREB, confirmed that a 4-d treatment with spadin (i.p.,10^−5^ M) stimulated the hippocampal phosphorylation of CREB, whereas the amount of total CREB remained unchanged (Figure 7F). Quantification of blots indicated a significant 4-fold stimulation of pCREB within the hippocampus extracts. To examine the relationship between pCREB and neurogenesis, expression of pCREB in newborn cells, visualized by DXC labeling, was examined by immunofluorescence. Double labeling for pCREB and DCX demonstrated a colocalization of pCREB expression in several precursor neurons in the presence of spadin (Figure 7G). These data pointed out that spadin induced a specific and rapid onset of neurogenesis and CREB activation in adult brain mice.

## Discussion

This study identifies spadin as the first peptidic antagonist of the TREK-1 channel and illustrates its potent antidepressant properties by using biochemical, electrophysiological, and behavioral approaches. Spadin is a partial peptide derived from the propeptide released from the precursor form of NTSR3/Sortilin. Here, we show for the first time that the propeptide is present into blood circulation and is able to inhibit currents mediated by the TREK-1 channel. Due to higher affinity and for a better efficacy, this study was mainly focused on the use of spadin. Using TREK-1 deficient mice and animal models of depression, our laboratory has recently identified the TREK-1 channel as a new target for depression and its blockers as potential antidepressant drugs [1]. With the identification of spadin as an antagonist of TREK-1, this work validates the TREK-1 channel as a good target for the development of drugs for the treatment of depression [1],[40]. In humans, the Star*D study has reported the functional role of this particular potassium channel in mood regulation and in resistance to antidepressant treatments [4], strengthening the idea that TREK-1 represents an attractive pharmacological target for the development of new types of antidepressant drugs.

This is of high relevance since depression is a devastating illness that affects ∼17% of the population at some point in life, resulting in major social and economic consequences [41]. Designing effective treatments for this serious disorder is challenging, in part because unraveling the exact changes that lead to this psychiatric disorder is particularly difficult. In addition to the inherent complexity of the disease itself, it is not clear how antidepressant drugs work. Most antidepressants increase levels of the monoamine serotonin (5-HT) and/or noradrenaline (NA), suggesting that biochemical imbalances within the 5-HT/NA systems may underlie the pathogenesis of this disorder [17],[27],[39]. To date, the mainstay of antidepressant treatments is constituted by SSRIs, which inhibit the 5-HT reuptake pump. Although antidepressant treatments significantly improve the therapeutic outlook for depressed patients, there are still too many patients who do not respond to initial treatments. In the case of response, side effects are often observed, as well as a delay in the onset of therapeutic efficiency and/or a partial rather than a full remission. Spadin, which is a natural peptide, may alleviate these problems and become a strong candidate to develop new efficient and fast-acting antidepressant treatments. The first result of this work is the identification of the NTSR3/Sortilin receptor as a novel TREK-1 partner protein. It interacts physically and functionally with TREK-1 to modify its cell surface expression. NTSR3/Sortilin is a member of the Vps10p-domain receptor family, which is expressed in several tissues, including the brain. The interaction between NTSR3/Sortilin and the N-terminal portion of the precursor form of the NGF (pro-NGF) and the brain-derived neurotrophic factor (pro-BDNF) represents a key event in the process that controls neurotrophins-mediated cell survival and death in developing neuronal tissue and post-traumatic neuronal apoptosis [42]. NTSR3/Sortilin is involved in the sorting of BDNF [43]. In regard to depression (for review see [44]), it is well known that exogenous delivery of neurotrophic factors, such as BDNF and/or neurotrophin 3 (NT-3) promotes the function, sprouting, and regrowth of 5-HT neurons in the rat brain. Infusions of BDNF into the DRN produced an antidepressant effect, as evaluated by several learned helplessness paradigms. Environmental stressors induce depression and decrease BDNF mRNA, whereas antidepressants increase BDNF mRNA in the brain via 5-HT_2A_ and β-adrenoreceptor subtypes. Since we observed an activation of 5-HT neurons by spadin, it would be important to measure the influence of spadin both on protein and mRNA levels of BDNF in order to determine whether its action is correlated with the modulation of neurotrophin pathways. NTSR3/Sortilin and spadin interact with the TREK-1 channel as shown by immunoprecipitation of TREK-1 and NTSR3/Sortilin from COS-7 cells co-expressing both proteins. TREK-1 and NTSR3/Sortilin are also colocalized in mouse cortical neurons.

This work identifies a new function for spadin as a peptidic antagonist of the TREK-1 channel. Until now, the full propeptide (1–44) which contains the active spadin was known to display two principal functions: (1) It binds the mature form of NTSR3/Sortilin, hindering ligands to access the binding site of the receptor, and (2) it antagonizes the effects of NT on microglial cell migration [9],[14]. To our knowledge, the propeptide has no additional protein target. Here, we determined that spadin, but not its N-terminal fragment Gln1-Arg16, displays identical binding and activity properties as those of the full propeptide on the NT system. Spadin binds specifically to TREK-1 with an affinity of 10 nM. Electrophysiological studies show that spadin efficiently blocks the TREK-1 activity in COS-7 cells, cultured pyramidal neurons, as well as CA3 hippocampal neurons in brain slices of wild-type mice and not in *kcnk2* ^−/−^ mice, suggesting a specific effect of spadin on the TREK-1 channel.

Finally, our data point out spadin as the first peptidic and fast-acting antidepressant. Considering the blocking effect of spadin on TREK-1 channels, we have analyzed in vivo its potential antidepressant effects. In behavioral tests (FST, TST, and CMST), predicting an antidepressant response [24], spadin-treated mice show a resistance to depression as do *kcnk2* ^−/−^ mice [1]. This antidepressant phenotype is even more marked in the LH and NSF tests, which are considered as classical “rodent models of depression” [13],[31]. The antidepressant effect of spadin is not specific to mice since it has also been observed in rats using the FST test and in vivo 5-HT neuron firing recordings. More importantly, our results indicate that molecular, biochemical, and behavioral changes, that have previously been specifically linked to long-term chronic treatment with SSRIs [45],[46], are already present as soon as 4 d when using i.v. spadin administration. The fast-acting antidepressant potential of spadin, observed in vivo in FST and TST tests, is further confirmed by its ability to activate CREB function and neurogenesis in the adult mouse hippocampus after a subchronic treatment. It is now well stated that antidepressants share the common property to positively modulate cellular growth and plasticity in mood-related brain areas. Indeed, CREB activity and neurogenesis are considered as specific markers of antidepressant action [47] but have never been observed before 2 wk of treatment when using classical antidepressants such as SSRIs. By binding to cAMP response element (CRE) sites, CREB mediates transcriptional responses to elevated levels of cAMP. CRE-mediated gene transcription is upregulated after chronic antidepressant treatment [48]. CREB upregulation activates downstream targets such as the brain-derived growth factor (BDNF) after antidepressant treatment by binding to CRE elements located in the promoter region of the BDNF gene [49]. Here, we show that a 4-d chronic treatment with spadin is able to enhance the pCREB/CREB ratio and consequently increases cell division and proliferation in the SGZ. In addition, the therapeutic potential of spadin appears to be specific of depression, in that it is unable to affect anxiety-related behaviors. This is in good agreement with the fact that TREK-1 deficient mice do not show an anxiety-resistant phenotype [1]. In contrast, both spadin and the deletion of the TREK-1 channel induce an hypoactivity of the HPA axis when animals are exposed to stress.

As described in *kcnk2* ^−/−^ mice [1], spadin leads to an in vivo increase in efficacy of 5-HT neurotransmission as evidenced by an increased firing activity of DRN 5-HT neurons. Even if the involvement of other aminergic systems in the pathophysiology of depression is certainly non-negligible, it remains that the facilitation of central 5-HT transmission constitutes the common property of all the antidepressant strategies, which have proved their efficiency. From a mechanical point of view, 5-HT_1A_ autoreceptor stimulation reduces DRN 5-HT neuronal firing and, consequently, 5-HT neurotransmission [28]. Inhibition of adenylate cyclase and activation of G-protein-coupled inwardly rectifying K+ channels (GIRK) are involved in this negative feedback [40]. The decrease in cAMP concentration (as a result of reduced adenylate cyclase activity) in 5-HT neurons is also thought to induce TREK-1 opening because of a consequent reduction of phosphorylation of Ser333 by PKA [16]. According to this model, spadin would induce a depolarization by closing TREK-1 channels and, as described for TREK-1 deficient mice [40], would therefore reduce the negative feedback on 5-HT neurons, resulting in increased 5-HT neurotransmission and in turn in antidepressant-like effects. Direct inhibition of TREK-1 by spadin may also contribute to enhanced 5-HT neuron excitability. Because (1) sortilin is the partner protein of the TREK-1 channel and (2) both proteins are colocalized in 5-HT-enriched areas known to be involved in the pathophysiology of depression such as the prefrontal and cingulate cortice, amygdala, hippocampus, nucleus accumbens, dorsal raphe, and hypothalamus [13], one may infer that spadin acts predominantly through a modulation of the brain 5-HT circuitry. Nevertheless, we cannot exclude that it can also involve other neurotransmission systems. Whatever the effector pathways though, the fact that spadin has no effects on *kcnk2* ^−/−^ mice indicates that its action is first and foremost mediated by a modulation of TREK-1 channels.

**Figure 8:**
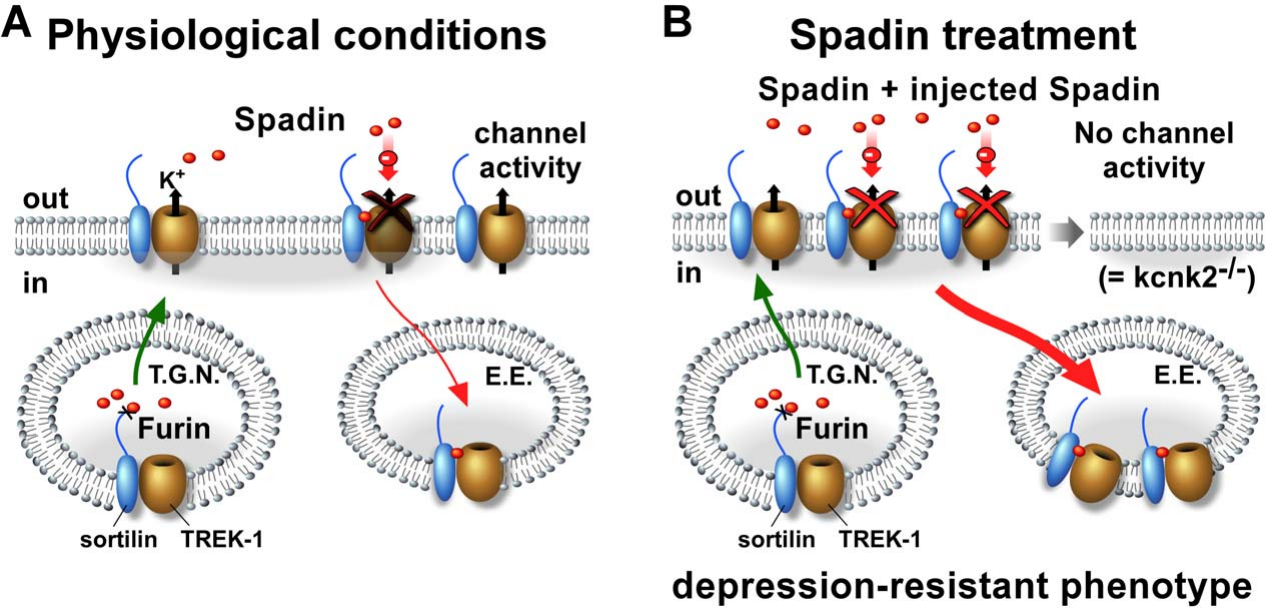
Schematic model of TREK-1 regulation by NTSR3/Sortilin and Spadin. In physiological conditions (A) the concentration of spadin that would be released from vesicles of the Trans Golgi Network (TGN) is not sufficient to completely abolish the channel activity, by internalization *via* Early Endosome (E.E.) vesicles, direct blockade, or both. Conversely, under spadin treatment (B) the amount of spadin is sufficient to internalize all channel molecules and consequently to abolish the channel activity.

Our results show that spadin induces an 80% internalization of these channels. We propose a model of regulation of TREK-1 expression and regulation by NTSR3/Sortilin receptor and spadin (Figure 8). In physiological conditions (Figure 8A), TREK-1 and NTSR3/Sortilin would associate in the TGN vesicle, where spadin is hydrolyzed by furin. When TGN vesicles merge to the plasma membrane, spadin would be released (as suggested in [14] and shown in Figure 2B–C and Figure S3) and would bind either NTSR3/Sortilin, TREK-1 channel, or both. This would lead to the internalization of the TREK-1/Sortilin complex in early endosome and subsequently to its degradation. In the presence of an excess of spadin given by administration (Figure 8B), the rate of internalized complexes would be increased, resulting in a total disappearance of TREK-1 channels at the surface membrane. This prediction is supported by the fact that 80% of the ^125^I-spadin bound on the TREK-1 transfected COS-7 cells are insensitive to an acid-NaCl wash (Figure 1F), indicating that the complex spadin-TREK-1 is already internalized. The consequent loss of TREK-1 channel activity would lead to an antidepressant phenotype, as observed in TREK-1 deficient mice. However, overall the inhibitory action of spadin on TREK-1 function is likely the consequence of both its ability to induce channel internalization and its direct effect on the channel current.

### Conclusions

Spadin can be considered as a natural endogenous antidepressant and constitutes the first peptide identified as an antidepressant with a rapid onset of action. Due to these peculiar properties, spadin brings a new concept to address the treatment of depression. To date, spadin is also the first blocker of TREK-1 channel identified, which not only is of relevance in the field of depression but also constitutes a useful tool to further understand the role of TREK-1 channels in other neurological pathologies. Finally, this work shows the development of a reliable method for dosing the propeptide and spadin by using AlphaScreen technology. The last point is crucial to use in the future spadin as a marker of depression by dosing spadin in the serum of depressed patients, and to help for setting clinical preventive protocols. Detecting and preventing the depression certainly could decrease the economic burden of this disease, which is estimated to be $44 billion per year in the United States.

## Materials and Methods

All experiments were carried out on 20–25 g male C57Bl/6J (Janvier France Breeding) and on TREK-1 deficient mice (*kcnk2* ^−/−^) according to policies on the care and use of laboratory animals of European Community legislation 86/609/EEC. The local Ethics Committee (CREEA Côte d'Azur) approved the experiments (protocol number NCE2008-08/09-0). The behavioral protocols are described in the Supporting File (Text S1).

### Drugs and Chemicals

The propeptide named spadin, with the following amino acid sequence, Y-APLPRWSGPIGVSWGLR (GenBank NM_019972 for mouse), and the N-terminal fragment QDRLDAPPPPAAPLPR were synthetized by Gencust (France). The stock solution was dissolved in distilled water at a concentration of 10^−3^ M, and spadin solution was then diluted in NaCl 0.9% to reach the different treatment concentrations. The i.c.v. injection was performed under isoflurane anesthesia. Mice were anesthetized by inhalation of 2% isoflurane mixed with 30% oxygen and 70% nitrous oxide. Spadin (5 µl) was stereotaxically administered 30 min prior to the behavioral test by an injection needle that was lowered bilaterally into the lateral ventricle of the mouse positioned on a stereotaxic frame, by using the coordinates referred from Paxinos and Flanklin (related to bregma: AP: −0.46 mm, ML: 1.25 mm, and DV: −2.25 mm). The injection needle was connected to a Hamilton syringe (10 µl) positioned in a micropump and delivered the drug solution at a rate of 1 µl/min for 5 min. Fluoxetine (TEVA Santé, France) and diazepam, diluted in NaCl 0.9%, were used at the concentration of 3 mg and 0.5 mg per kg body weight, respectively, in i.p. administration. BrdU (Sigma-Aldrich, St Quentin Fallavier, France) was diluted in Tris-buffered saline (0.1 M in NaCl 0.9%, pH:7.6). All other chemicals were from Sigma (St Quentin Fallavier, France). Stock solutions were prepared in H_2_O except otherwise mentioned, frozen, and diluted before the experiment. Arachidonic acid that was prepared at a concentration of 0.1 M under argon in 100% ethanol, glibenclamide 100 mM in dimethyl sulfoxide (DMSO), and fluoxetine 1.3 mM in glycerol.

### Cell Culture

COS-7 and C13NJ cells were cultured in DMEM supplemented with 10% FBS and 50 µg/ml gentamycin at 37°C under 5% CO2. β-TC3 cells were grown in RPMI 1640 supplemented with 2.5% FBS, 50 µM β-mercaptoethanol, 10 mM HEPES, 1 mM Sodium pyruvate, and 50 µg/ml gentamycin. Cells were maintained at 37°C under 5% CO2.

Cortical neurons were prepared from 14 old mouse embryos, whereas hippocampal neurons were prepared from newborn mice. Briefly, dissected brain areas were dissociated and neurons were plated on polylysine-treated 35 or 60 mm dishes and maintained in culture in Neurobasal medium supplemented with B27, Glutamax, and antibiotics for 2 to 3 wk before to be used for electrophysiological or biochemical experiments.

#### Characterization of the interaction between NTSR3/Sortilin, spadin, and TREK-1

Experiments were performed using COS-7 cells (10^6^ cells per diameter 100 dish) transfected with TREK-1 (2 µg/dish) in the presence or in the absence of NTSR3/sortilin (2 µg/dish) using the DEAE-dextran protocol.

#### Immunoprecipitation

Cortical neurons or COS-7 cells transfected with TREK-1 and NTSR3/Sortilin were lysed in 20 mM Tris-HCl pH 7.5, 50 mM NaCl, 50 mM NaF, 30 mM sodium pyrophosphate, 5 mM EGTA, 10% glycerol, 1% Triton X100, 1 mM PMSF, 1 mM Na3VO4, and 5 µg/ml aprotinin (lysis buffer) for 1 h at 4°C. Solubilized proteins were clarified by centrifugation at 15,000×g for 15 min at 4°C. Supernatants were immunoprecipitated by using either the rabbit polyclonal anti-NTSR3 antibody (1∶250) (Alomone) or the rabbit polyclonal anti-TREK-1 antibody (1∶250) (Alomone) in the presence of 40 µl protein-A Affarose (Interchim) overnight at 4°C. Protein complexes were recovered by centrifugation at 15,000×g for 5 min at 4°C and washed twice with the lysis buffer. Immunoprecipitates were resuspended in SDS buffer, separated by SDS-PAGE, transferred onto nitrocellulose, and revealed either with anti-NTSR3 or with anti-TREK-1 (1∶1000). Bound antibodies were visualized using HRP-conjugated goat anti-rabbit IgG TrueBlot.

#### Sub-cellular fractionation

Plasma membranes were prepared from COS-7 cells transfected with TREK-1 alone or with NTSR3/Sortilin according to the protocol described by Clancy and Czech [50]. 30 µg of crude homogenates or purified plasma membranes were submitted to Western blot analysis using the rabbit polyclonal anti-TREK-1 antibody (1∶500). Alternatively, plasma membranes of COS-7 cells transfected with TREK-1 alone or with NTSR3/Sortilin were labeled with 0.5 mg/ml Sulfo-NHS-SS-Biotin for 30 min at 4°C. Cells were recovered with the lysis buffer used for immunoprecipitation for 1 h at 4°C and solubilized proteins were clarified by centrifugation at 15,000 g for 15 min at 4°C before to be precipitated using streptavidin-agarose overnight at 4°C. Protein complexes were recovered, separated by SDS-PAGE, and submitted to Western blot analysis as described above.

#### Spadin iodination

Spadin (2 nmol) was iodinated with ^125^INa (0.5 nmol) using lactoperoxidase as oxidant. Monoiodinated spadin (on Tyr0) was purified by HPLC using a Waters apparatus equipped with a RP18 Lichrosorb column. Elution was carried out at a flow rate of 1 ml/min with a linear gradient of increasing concentration of acetonitrile in water containing 0.1% TFA from 30% to 60% in 36 min. The iodinated peptide was eluted at 24 min.

#### Binding assays

For competition experiments, homogenates from TREK-1 transfected COS-7 or C13NJ cells were incubated with 0.2 nM ^125^I-spadin or ^125^I-NT (200,000 cpm in 250 µl) iodinated and purified as previously described [51]. Incubations were performed in 50 mM Tris-HCl, pH 7.4 containing 0.1% BSA in the presence of increasing concentrations of non-radioactive spadin or NT (10^−10^ to 10^−5^M). Incubations were terminated by addition of 2 ml of ice-cold binding buffer followed by filtration through cellulose acetate filters (Sartorius, Göttingen, Germany) and washing twice with 2 ml of ice-cold buffer. Radioactivity on filters was counted with a gamma-counter.

#### Wound-healing assay

A cell-free zone was created within a semi-confluent monolayer of microglial culture by scratching cells off with a pipette tip. We analyzed by time-lapse microscopy how cells repopulated the cell-free zone, as already described [17].

#### Internalization

Cells, grown on 12 mm multiwell-dishes, were incubated with 0.2 nM ^125^I-spadin for various times at 37°C in an Earle's Tris-Hepes buffer (cell binding buffer). Incubations were terminated by washing cells twice with the binding buffer or with the same buffer containing 0.5 M NaCl, pH 4, for 2 min to remove non-sequestrated radioactivity (acid-NaCl wash). Cells were harvested with 1 ml of 0.1 N NaOH and counted in a gamma-counter. Non-specific binding was determined in the presence of 10 µM unlabeled spadin.

#### TREK-1/NTSR3/Sortilin colocalization experiments

Hippocampal neurons were first washed for 5 min in Phosphate-Buffered Saline (PBS), then fixed with 4% paraformaldehyde in PBS for 20 min at room temperature. Coverslips were rinsed twice with PBS and incubated with 50 mM NH4Cl in PBS for 10 min to quench excess of free aldehyde groups. After 20 min in PBS containing 10% Horse Serum (HS), cells were labeled with a goat polyclonal anti-NTSR3/Sortilin (1/100) (Santa Cruz) and a rabbit anti-TREK-1 [16] (1/3,000), for 1 h at room temperature in PBS containing 5% HS. Cells were rinsed three times in PBS, then incubated at room temperature in PBS containing FITC conjugated donkey anti-goat antibody (1/1,000) and a Texas Red conjugated donkey anti-rabbit antibody (1/1,000) in PBS containing 5% HS for 45 min. After two washes with PBS and one with water, coverslips were mounted on glass slides with mowiol for confocal microscopy examination.

### Electrophysiology

#### COS-7 cells

All electrophysiological experiments were done on COS-7 cells seeded at a density of 20,000 cells/35-mm dish 24 h before transfection. Cells were transfected by the classical DEAE-dextran method with TREK-1-GFP plasmids (0.1 µg/µL). Cells were visualized 48–72 h after transfection using fluorescence.

The whole-cell patch-clamp technique was used to evaluate TREK-1 potassium channel current by using a RK 400 patch-clamp amplifier (Axon Instruments, USA), as previously described [52]. Currents were lowpass filtered at 3 kHz, digitized at 10 kHz using a 12-bit analog-to-digital converter. Patch-clamp pipettes were pulled from borosilicate glass capillaries and had a resistance of 1.8–3 MÙ. The bath solution contained (in mM) 150 NaCl, 5 KCl, 3 MgCl_2_, 1 CaCl_2_, and 10 HEPES, adjusted to pH 7.4 with NaOH; the patch pipette solution contained (in mM) 155 KCl, 3 MgCl_2_, 5 EGTA, and 10 HEPES, adjusted to pH 7.2 with KOH. Cells were clamped at −80 mV and voltage changes were either applied by ramp (from −100 to 50 mV, 1 s in duration) or by step (from −100 to 40 mV, 1.5 s in duration). Cells were continuously superfused with a microperfusion system. The pipette capacitance was not subtracted from total membrane capacitance and there was no leak subtraction. All experiments were done at room temperature (21°C to 22°C) and in the presence of a cocktail of potassium channel inhibitors (K^+^ blockers: 10 mM tetraethyl ammonium (TEA), 3 mM 4-aminopyridine (4-AP), 50 nM charybdotoxin, 10 µM glibenclamide, 100 nM apamin).

Pclamp software was used to analyze currents recorded in the whole-cell mode measured at 0 mV. Results are expressed as means ± SD. To obtain the IC_50_ value for dose-dependent inhibition, experimental data were fitted with a standard sigmoidal function.

#### β-TC3 cells

Native currents elicited by these cells were recorded in the whole-cell configuration of the patch-clamp technique (as described for COS-7 cells) and in the presence of K^+^ blockers.

#### Brain slices of hippocampus

12–27-d-old mice were anaesthetized with 1% halothane. Following decapitation, brains were rapidly removed and placed in cold phosphate/bicarbonate buffered solution (PBBS, 4°C) composed of (mM) 125 NaCl, 2.5 KCl, 0.4 CaCl_2_, 1 MgCl_2_, 25 glucose, 1.25 NaH_2_P0_4_, 26 NaHC0_3_, and pH 7.4 when bubbled with 95% O_2_/5% CO_2_. Transversal 250 µm thick hypothalamic slices cut with a vibrating microtome (Microm, Francheville, France) were then transferred to an incubation chamber maintained at 34°C in oxygenated PBBS. After 1 h, slices were transferred to another incubation chamber at room temperature (22–25°C) filled with PBBS containing 2 mM CaCl_2_.

For current measurements using the whole-cell patch-clamp technique, brain slices were placed under a Nomarski microscope (Zeiss, Le Pecq, France) equipped with infrared video camera (Axiocam, Zeiss, Le Pecq, France) in a recording chamber superfused at a flow rate of 1 ml.min^−1^ with HEPES solution containing (in mM) 140 NaCl, 5 KCl, 2 CaCl_2_, 2 MgCl_2_, 10 Glucose, 10 Hepes, and pH 7.4. Pictures were taken by using a digital camera (Axiocam, Zeiss) connected to image-acquisition software (Axiovision). Recordings were made at room temperature (25°C±2°C) using an Axopatch 200B (Axon Instruments, Foster City, CA, USA). Patch-clamp pipettes made from borosilicate glass capillary (Hilgenberg, Masfeld, Germany) had a resistance of 4–10 MΩ when filled with the internal solution containing (mM) 135 KCl, 5 NaCl, 2 MgCl_2_, 5 EGTA, and 10 Hepes (pH adjusted to 7.25 with KOH). Values of access resistance ranged from 12 to 20 MΩ and were not compensated. Measurements were made 2–3 min after obtaining the whole-cell to ensure dialysis. Changes of extracellular solution were obtained by a fast multi-barrel delivery system positioned close to the cell tested. Stock solutions were prepared in H_2_O except otherwise mentioned, frozen, and diluted before the experiment. Arachidonic acid, which was prepared at a concentration of 0.1 M under Argon in 100% ethanol, glibenclamide 100 mM in dimethyl sulfoxide (DMSO), and fluoxetine 1.3 mM in glycerol.

Statistical significance between groups (average data expressed as mean ± SEM, n = number of neurons) was tested using the Student's *t* test or the ANOVA followed by t test, and were considered significant at *p*<0.05. Statistical analysis was done using SigmaPlot (Jandel) and Origin (Microcal) softwares.

#### Extracellular unitary recordings of DRN 5-HT neurons

As previously described [1], single-barreled glass micropipettes (recording electrodes) were filled with a 2 M NaCl solution saturated with Fast Green FCF, resulting in an impedance of 2–5 MΩ. Mice were anaesthetized with chloral hydrate (400 mg/kg, i.p., using a 2% solution) and placed in a stereotaxic frame equipped with the Stoelting “just for mouse” adaptor. Electrodes were positioned 0.5–1 mm posterior to the interaural line on the midline and were then lowered into the DRN, usually attained at a depth of 2.5 mm from the brain surface. 5-HT neurons were then encountered over a maximal distance of 1 mm. They were identified using the following criteria: a slow (0.5–2.5 Hz) and regular firing rate and long-duration (0.8–1.2 ms) action potentials, with a positive-negative spike [1]. Spikes were computed by using the Spike 2 software, so that the firing rate was calculated as the mean number of events occurring within a 10 s period. For each neuron, the discharge was monitored during 60 s. Each mouse received either spadin (10^−5^ M in a 100 µl bolus) or its vehicle (saline). Starting 30 min after the injection, 3 to 4 successive descents were performed along the DRN, for a total of 4–8 cells recorded per animal. Recordings were performed for a maximal duration of 4 h post-injection.

### Measurement of Hippocampal Neurogenesis

Twenty-four hours after the injection of BrdU (120 mg per kg of body weight in a 300 µl bolus), mice were euthanized and transcardially perfused with 4% cold paraformaldehyde. Serial sections of the brains were cut (40 µm) throughout the entire hippocampus on a vibratome (Leica). Every sixth section throughout the hippocampus was processed for BrdU or DCX immunohistochemistry, as previously described [1]. For each immunodetection, slides were first incubated overnight at 4°C with a mouse monoclonal anti-BrdU antibody (1∶200; Becton-Dickinson) or a goat anti-DCX (1∶400; Santa Cruz Laboratories). For chromogenic immunodetection, sections were then incubated for 1 h in biotin-conjugated species-specific secondary antibodies (1∶100; Vector Laboratories) followed by a peroxidase-avidin complex solution according to the manufacturer's protocol. The peroxidase activity of immune complexes was visualized with DAB staining using the VectaStain ABC kit (Vector Laboratories). For fluorescent double-labeling, performed to determine the cell phenotype, sections were incubated overnight at 4°C with anti-sheep BrdU (1∶200, Interchim), anti-goat DCX (1∶200, Santa Cruz Laboratories), or an anti-GFAP (glial fibrillary acidic protein, marker for astrocytes, 1∶250, Dako). Antibodies were revealed with anti-IgG Alexa 488 or 594-coupled antibodies (1∶400; Molecular Probes). All BrdU-labeled cells in the granular cell layer and SGZ were counted in each section (*n* = 10 and 5 mice per group) at 400× and 1,000× magnification under a light microscope (Olympus) by an experimenter blinded to the study code. The total number of BrdU-positive cells per section was multiplied by 6 to obtain the total number of cells per dentate gyrus. The counting of BrdU/DCX labeled cells was performed using a Laser Scanning Confocal Microscope (TCS SP, Leica) equipped with a DMIRBE inverted microscope.

### Assessment of CREB Activation

#### Total CREB and pCREB Western blotting

Mouse brains were dissected on ice. Isolated hippocampi (1–2 mg wet tissue/100 µl) were homogenized in a solubilization buffer containing 20 mM HEPES (pH:7.9), 0.4 M NaCl, 20% (v/v) glycerol, 1% (v/v) Nonidet P-40, 5 mM MgCl_2_, 0.5 mM EDTA, 0.1 mM EGTA, and protease inhibitor cocktail using a dounce homogenizer. The homogenates were centrifuged 30 min at 15,000×g at 4°C. Supernatants were stored at −70°C until further use. Protein concentrations were measured using conventional Bradford's method. Samples (50 mg proteins from each experimental group) were resuspended in SDS buffer, sonicated, boiled for 5 min before loading on 10% SDS PAGE gels, and electrophoresed for 2 h at 60 mA. Proteins were then transferred from gels onto Hybond-PVDF membranes (Amersham Biosciences) in blotting buffer (156 mM Tris, 1 M glycine, PBS) for 90 min at 80 mA and blocked with 5% skim milk (Regilait) in PBS for 2 h at room temperature. Membranes were incubated with the monoclonal anti-pCREB antibody (1∶1,000, Cell Signaling Technology) overnight at 4°C. Total CREB or tubulin contents were determined after stripping by using 1/1,000 dilution of anti-CREB antibody (Cell Signaling Technology) and 1/1,000 dilution of anti-tubulin antibody (Sigma-Aldrich). After washing in 0.1% Tween/PBS (four times, 15 min each), secondary anti-mouse or anti-rabbit horseradish peroxidase-conjugated antibody (Amersham Biosciences, 1/10,000) was used for 1 h at room temperature. Detection of blotted proteins was performed using ECL plus Western blotting detection reagents (Amersham Biosciences, Orsay, France) with a Las-3000 imaging system (Fujifilm). Relative intensities of the labeled bands were analyzed by densitometric scanning using ImageJ imaging system. CREB activation was expressed as the ratio between pCREB and total CREB present in each sample.

#### pCREB immunostaining in hippocampal sections

Free floating sections were permeabilized in 0.1% Triton/PBS for 10 min and blocked with 3% goat serum/PBS for 2 h at room temperature followed by a single wash in PBS. Sections were then incubated with the monoclonal anti-pCREB antibody (1∶300, Cell Signaling Technology) overnight at 4°C. After three washes in PBS, sections were then incubated in biotinylated horse anti-rabbit IgG (Jackson ImmunoResearch, diluted 1∶15,000) for 2 h at room temperature. pCREB expression was visualized by 3.3′-diaminobenzidine (DAB) staining using the VectaStain ABC kit (Biovalley). All sections were washed and mounted with Entellan. For BrdU/pCREB immunofluorescence staining, free floating sections of mice injected with BrdU were incubated with the primary antibodies, anti-rabbit pCREB (1∶500, Cell Signaling Technology), and anti-mouse BrdU (1∶40, Becton-Dickinson). After three washes in PBS, sections were then incubated in anti-IgG Alexa 488- or 594-coupled antibodies (Molecular Probes) for 1 h at room temperature. After drying sections in the dark boxes for 2 h, they were mounted with Vectashield (Vector Laboratories). Confocal microscopy observations were performed with the Laser Scanning Confocal Microscope (TCS SP, Leica) equipped with a DMIRBE inverted microscope, using a Plan Apo 63×/1,4 NA oil immersion objective.

#### Corticosterone assay

Mice were individually housed for at least 12 h before blood sample collection. Serum samples, collected in the morning, were obtained by retroorbital puncture before and 30 min after the end of a 10 min tube restraint. Levels of corticosterone were measured by radioimmunoassay using a commercially available kit (MP Biomedicals).

## Supporting Information

Figure S1 **C13NJ wound-healing assay.** A cell-free zone was created within a semi-confluent monolayer with a pipette tip. We analyzed by time-lapse microscopy how cells repopulated the cell-free zone. The 100% of migrating cells was calculated by stimulation of cell migration with 10% of Fetal Calf Serum (FCS).
(0.13 MB TIF)

Figure S2 **Comparative effects of N-terminal propeptide fragment Gln1-Arg16 and spadin on TREK-1 channel activity: in COS-7 transfected cells.** After 90 s of application, the N-terminal propeptide fragment Gln1-Arg16 (Nt-Peptide 1-16) was unable to inhibit the current increase induced by a 10 µM arachidonic acid (aa) application (the current value continued to increase) (A), whereas in the same experimental conditions spadin inhibited the aa-induced increase (B).
(0.16 MB TIF)

Figure S3 **Alpha Screen assays.** Competition curve obtained with four groups (n° 2 to 5) of 6 mice. Values obtained are compared to the standard curve.
(0.20 MB TIF)

Figure S4 **Effects of spadin on cultured pyramidal neurons from hippocampus.** (A) Cultured pyramidal neuron from hippocampus, with on top, the patch-clamp pipette. Neurons were chosen according to their morphology. (B) Current recorded in response to ramps of potential obtained in the various conditions indicated (aa, arachidonic acid: 10 µM; Spadin: 1 µM; Fluoxetine: 20 µM). Neurons were recorded in the whole-cell configuration of the patch-clamp technique. In voltage-clamp, ramps of potential from −90 mV to 70 mV were applied every 10 s. The % variation of the current amplitude was always measured at −50 mV. The currents were recorded in response to such ramps in control conditions and in the presence of a cocktail of potassium blockers suitable to isolate the TREK currents (10 mM tetraethyl ammonium (TEA), 3 mM 4-aminopyridine (4-AP), 50 nM charybdotoxin, 10 µM glibenclamide, 100 nM apamin). In the presence of the potassium blockers, the remaining current was increased by about 50% by 10 µM arachidonic acid in 7 out of 70 recorded neurons (B–C), suggesting the activation of a TREK current. This acid-arachidonic evoked current (putative TREK current) was 49.7%±16.38% (n = 6) blocked by 1 µM spadin and fully blocked by 20 µM fluoxetine (B–D). The blocks were reversible. (C) Time course of spadin and fluoxetine effects. Applications are indicated by the horizontal bars.
(2.03 MB TIF)

Figure S5 **Antidepressant effect of spadin in rats.** (A, B) Effect of spadin on the average DRN 5-HT neuron firing rate. Spadin (10^−5^ M in a 500 µl bolus) or its vehicle (saline) was i.p. administered. Recordings started 30 min after the injection and were performed for a maximal duration of 210 min thereafter. (A) 5-HT neuron firing activity, calculated on the basis of all the cells recorded within the successive tracks performed along the DRN. Values at the bottom of each column indicate the total number of neurons recorded (n = 4 rats in both groups). In saline-injected rats, we found a value of 1.18±0.13 Hz, whereas it reached 2.66±0.36 Hz in the group treated with spadin (10^−5^M in a 500 µl bolus, i.p.) [one-way ANOVA, *F*(1, 68) = 13.06, *p*<0.001]. This effect corresponds to an increase of 125%, a value strikingly similar to that of 146% observed in the mice experiments. (B) Again, and as illustrated, several neurons found in spadin-injected rats discharged at up to 4, 5, or even 6 Hz, whereas most of the frequencies found in the saline group were in a normal (0.8–1.6 Hz) range. Samples of “descents” performed along the DRN, showing typical integrated firing rate histograms in a vehicle- (left panel), or in a spadin-treated (right panel), animal. Each cluster represents the electrical activity of one neuron, each bar representing the average number of recorded action potentials per 10 s. Insets, examples of action potential waveforms of 5-HT neurons. (C) Acute antidepressant effects of Spadin in Forced Swimming Test (FST). Spadin (10^−5^ M), Fluoxetine (20 mg/kg), or Saline solutions were injected 30 min before the test in rats (n = 10 per group). Spadin-treated rats had a shorter time of immobility comparable to those obtained in fluoxetine-treated animals (one-way ANOVA, F_2, 26_ = 16.66, ***p<0.001, **p<0.01 versus saline-treated rats).
(0.32 MB TIF)

Figure S6 **Mouse locomotion activity.** To determine whether spadin induced a change in locomotor activity, mice (n = 8 per group) were injected with the saline solution or spadin (10^−5^M in 100 µl bolus, i.p.) 30 min before starting the test session. Locomotor activity was monitored individually for 24 h using an infrared photobeam activity monitoring system (Imetronic, Pessac, France), which measured consecutive horizontal beam breaks. Testing was in transparent plastic cages (43×2 0×20 cm^3^) with fresh bedding in a grid of 8 cm horizontal infrared beams. Locomotor activity was defined as breaking of consecutive photobeams. Mice were given 1 h habituation session before being treated with spadin or saline. Movements were recorded and totalized for each 5 min period during the first hour and then by 10 min time sections for the next 23 h. Six periods were pooled to obtain data for 1 h of time. Different movements were monitored: the coming-and-going between the back and the front of the cage, climbing, and other movements in the back or the front of the cage. Data are the mean value of 8 animals per condition, bars represent SEM. Mice were kept under standard laboratory conditions: 12∶12 light-dark cycle with free access to food and water during the experiment. There was no significant difference in locomotor activity between spadin- and saline-treated mice within 1 or 24 h after the drug injection.
(0.53 MB TIF)

Text S1 **Supplementary material and methods.**
(0.07 MB DOC)

## Abbreviations

BDNF: brain-derived neurotrophic factor
BrdU: 5-bromo-2′deoxyuridine
CMST: Conditioned Suppression of Motility
CRE: cAMP response element
CREB: cAMP response element-binding
CS: conditioned suppression
DCX: doublecortin
DRN: Dorsal Raphe Nucleus
FCS: fetal calf serum
FST: forced swimming test
GFAP: glial fibrillary acidic protein
GIRK: G-protein-coupled inwardly rectifying K+ channels
HPA: hypothalamic-pituitary-adrenal
i.c.v.: intracerebroventricular
i.p.: intraperitoneal
i.v.: intravenous
LH: Learned Helplessness
NA: noradrenaline
NSF: Novelty Suppressed Feeding
NT: neurotensin
NT-3: neurotrophin 3
NTSR3: neurotensin receptor 3
proNGF: precursor of the Nerve Growth Factor
RAP: receptor-associated protein
SGZ: subgranular zone
SSRIs: selective serotonin reuptake inhibitors
TST: Tail Suspension Test
