# Supplementary figures and images for: Spadin, a Sortilin-Derived Peptide, Targeting Rodent TREK-1 Channels: A New Concept in the Antidepressant Drug Design
Source: PLoS Biol. 2010 Apr 13;8(4):e1000355. doi: 10.1371/journal.pbio.1000355 (PMC2854129; doi:10.1371/journal.pbio.1000355)

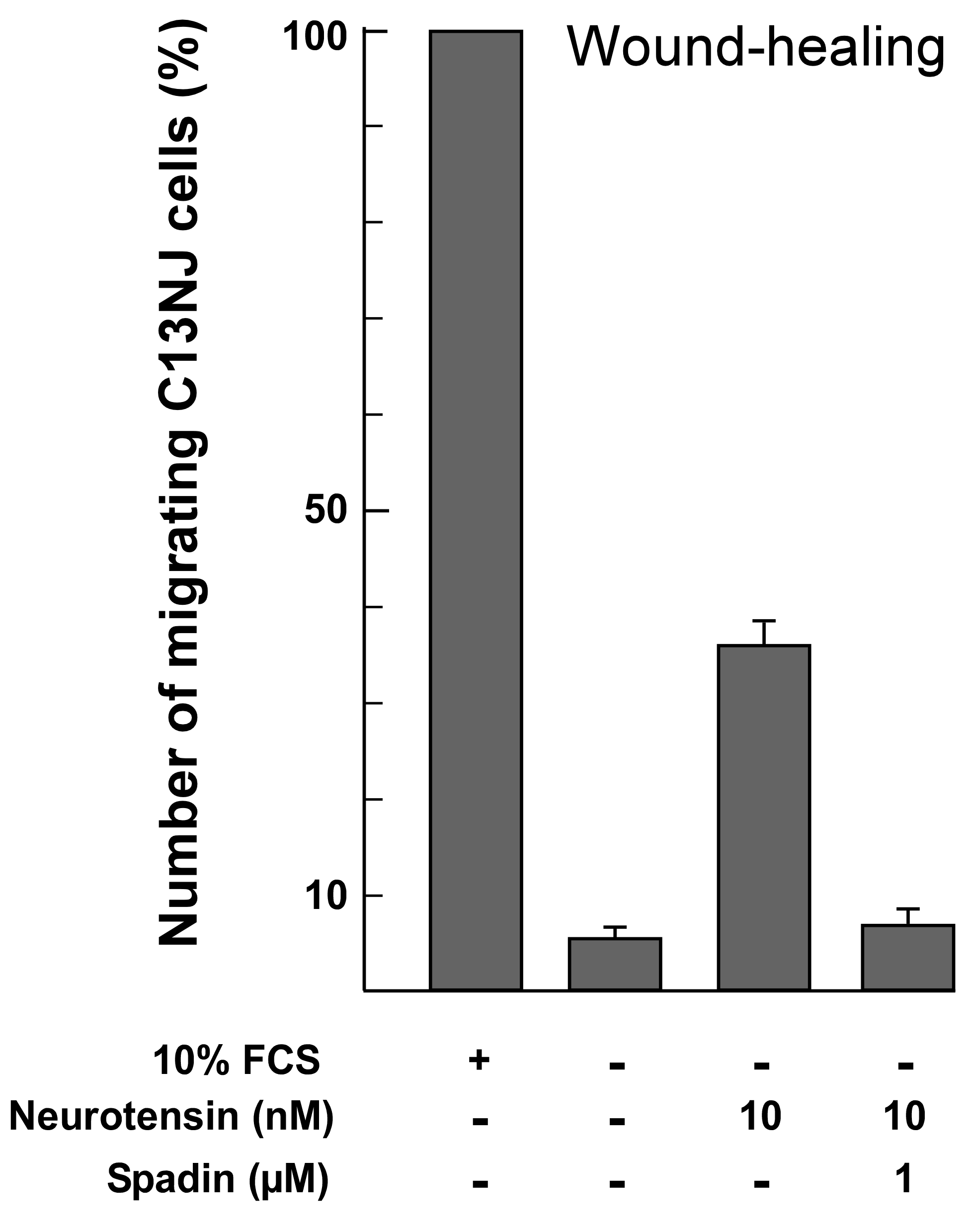

Supplement: Figure S1 — C13NJ wound-healing assay. A cell-free zone was created within a semi-confluent monolayer with a pipette tip. We analyzed by time-lapse microscopy how cells repopulated the cell-free zone. The 100% of migrating cells was calculated by stimulation of cell migration with 10% of Fetal Calf Serum (FCS). (0.13 MB TIF) [file pbio.1000355.s001.tif]

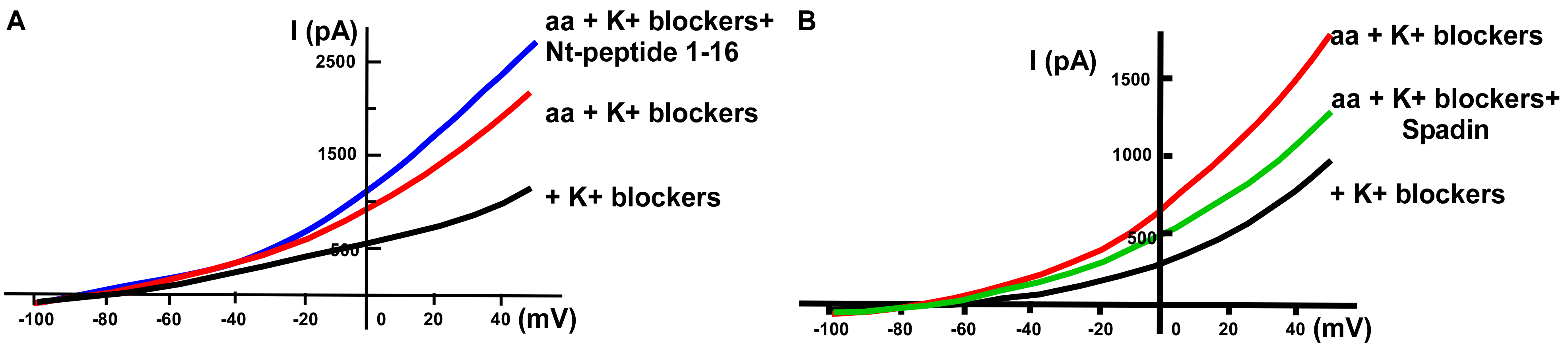

Supplement: Figure S2 — Comparative effects of N-terminal propeptide fragment Gln1-Arg16 and spadin on TREK-1 channel activity: in COS-7 transfected cells. After 90 s of application, the N-terminal propeptide fragment Gln1-Arg16 (Nt-Peptide 1-16) was unable to inhibit the current increase induced by a 10 µM arachidonic acid (aa) application (the current value continued to increase) (A), whereas in the same experimental conditions spadin inhibited the aa-induced increase (B). (0.16 MB TIF) [file pbio.1000355.s002.tif]

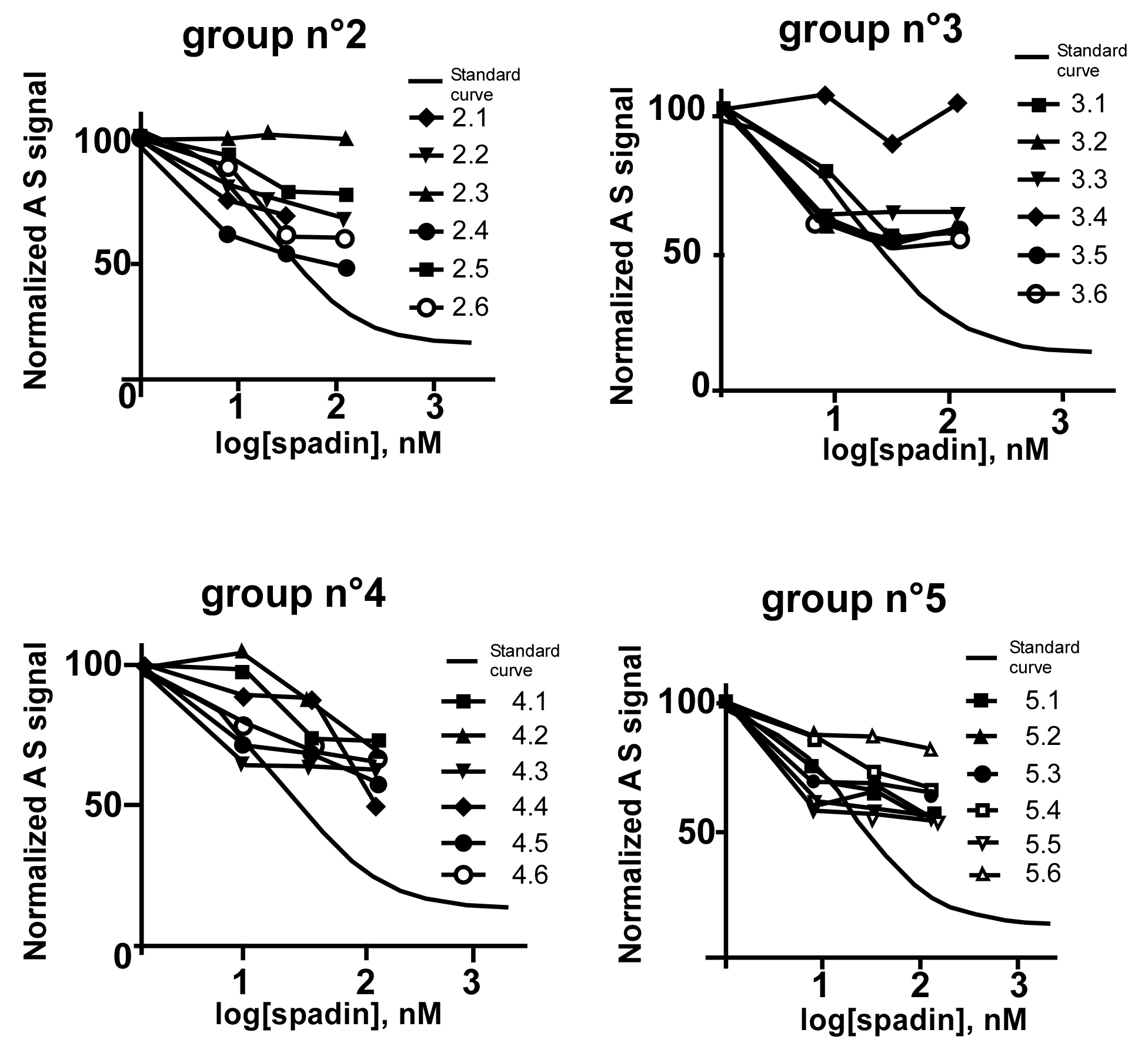

Supplement: Figure S3 — Alpha Screen assays. Competition curve obtained with four groups (n° 2 to 5) of 6 mice. Values obtained are compared to the standard curve. (0.20 MB TIF) [file pbio.1000355.s003.tif]

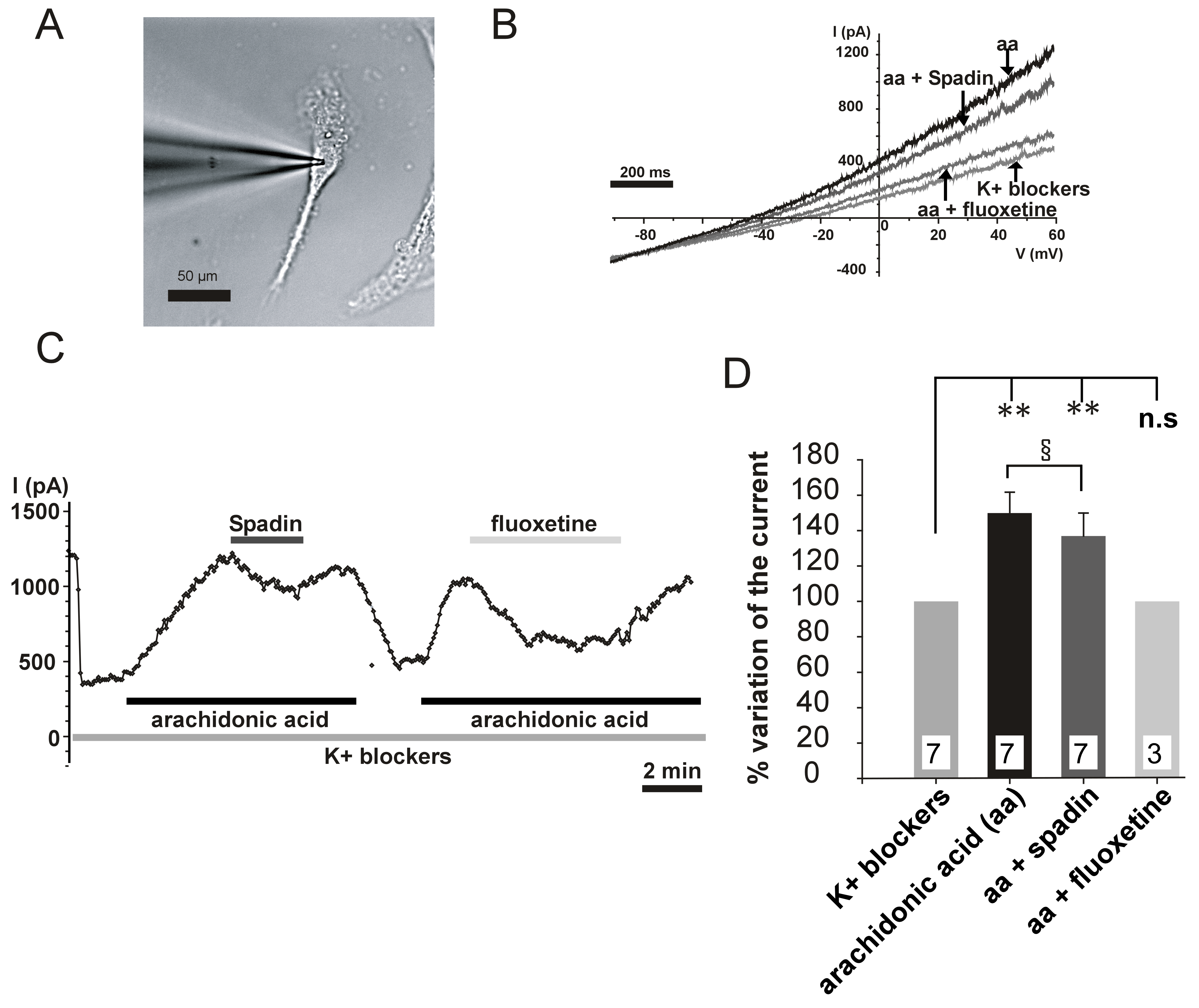

Supplement: Figure S4 — Effects of spadin on cultured pyramidal neurons from hippocampus. (A) Cultured pyramidal neuron from hippocampus, with on top, the patch-clamp pipette. Neurons were chosen according to their morphology. (B) Current recorded in response to ramps of potential obtained in the various conditions indicated (aa, arachidonic acid: 10 µM; Spadin: 1 µM; Fluoxetine: 20 µM). Neurons were recorded in the whole-cell configuration of the patch-clamp technique. In voltage-clamp, ramps of potential from −90 mV to 70 mV were applied every 10 s. The % variation of the current amplitude was always measured at −50 mV. The currents were recorded in response to such ramps in control conditions and in the presence of a cocktail of potassium blockers suitable to isolate the TREK currents (10 mM tetraethyl ammonium (TEA), 3 mM 4-aminopyridine (4-AP), 50 nM charybdotoxin, 10 µM glibenclamide, 100 nM apamin). In the presence of the potassium blockers, the remaining current was increased by about 50% by 10 µM arachidonic acid in 7 out of 70 recorded neurons (B–C), suggesting the activation of a TREK current. This acid-arachidonic evoked current (putative TREK current) was 49.7%±16.38% (n = 6) blocked by 1 µM spadin and fully blocked by 20 µM fluoxetine (B–D). The blocks were reversible. (C) Time course of spadin and fluoxetine effects. Applications are indicated by the horizontal bars. (2.03 MB TIF) [file pbio.1000355.s004.tif]

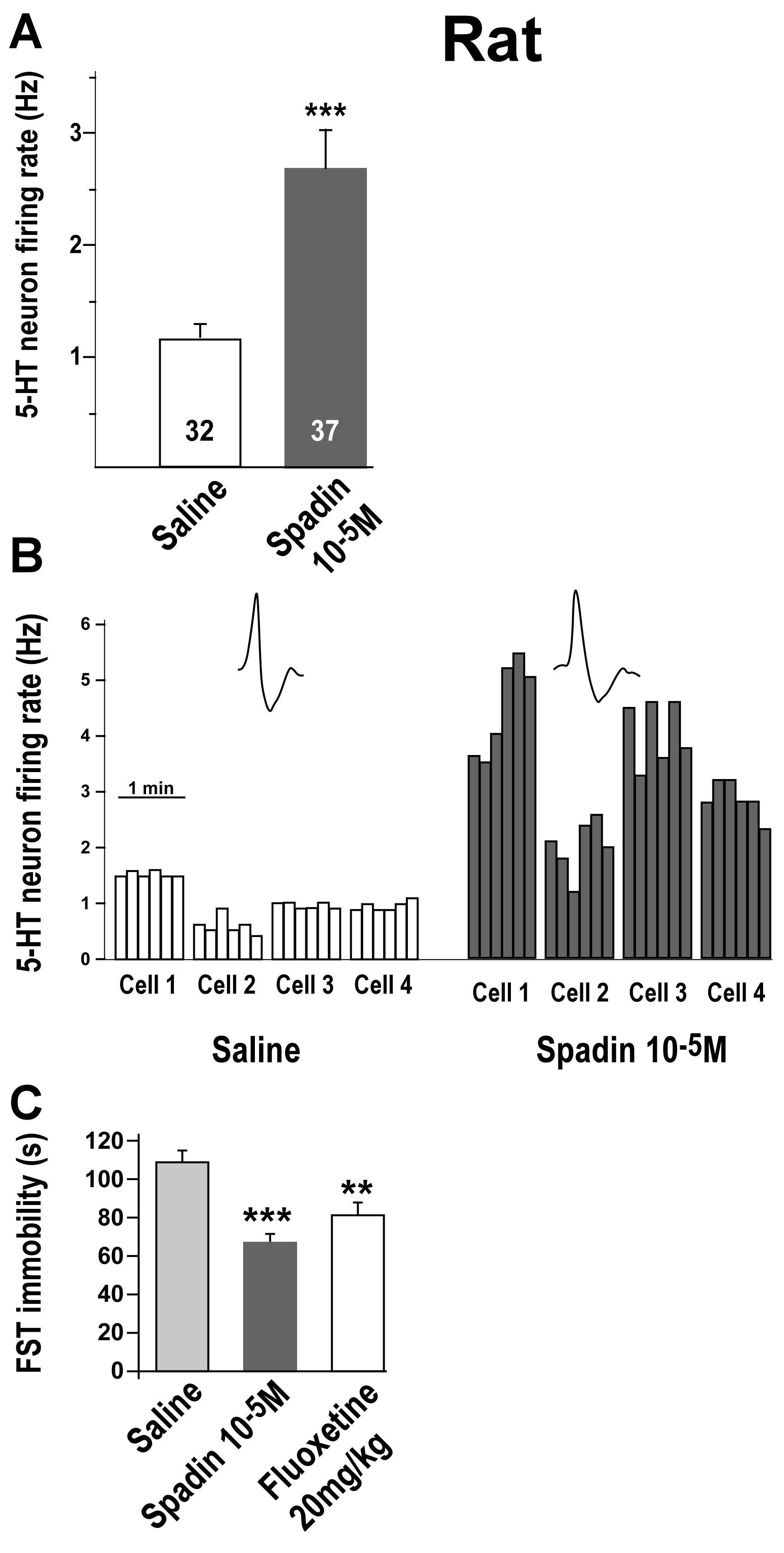

Supplement: Figure S5 — Antidepressant effect of spadin in rats. (A, B) Effect of spadin on the average DRN 5-HT neuron firing rate. Spadin (10−5 M in a 500 µl bolus) or its vehicle (saline) was i.p. administered. Recordings started 30 min after the injection and were performed for a maximal duration of 210 min thereafter. (A) 5-HT neuron firing activity, calculated on the basis of all the cells recorded within the successive tracks performed along the DRN. Values at the bottom of each column indicate the total number of neurons recorded (n = 4 rats in both groups). In saline-injected rats, we found a value of 1.18±0.13 Hz, whereas it reached 2.66±0.36 Hz in the group treated with spadin (10−5M in a 500 µl bolus, i.p.) [one-way ANOVA, F(1, 68) = 13.06, p<0.001]. This effect corresponds to an increase of 125%, a value strikingly similar to that of 146% observed in the mice experiments. (B) Again, and as illustrated, several neurons found in spadin-injected rats discharged at up to 4, 5, or even 6 Hz, whereas most of the frequencies found in the saline group were in a normal (0.8–1.6 Hz) range. Samples of “descents” performed along the DRN, showing typical integrated firing rate histograms in a vehicle- (left panel), or in a spadin-treated (right panel), animal. Each cluster represents the electrical activity of one neuron, each bar representing the average number of recorded action potentials per 10 s. Insets, examples of action potential waveforms of 5-HT neurons. (C) Acute antidepressant effects of Spadin in Forced Swimming Test (FST). Spadin (10−5 M), Fluoxetine (20 mg/kg), or Saline solutions were injected 30 min before the test in rats (n = 10 per group). Spadin-treated rats had a shorter time of immobility comparable to those obtained in fluoxetine-treated animals (one-way ANOVA, F2, 26 = 16.66, ***p<0.001, **p<0.01 versus saline-treated rats). (0.32 MB TIF) [file pbio.1000355.s005.tif]

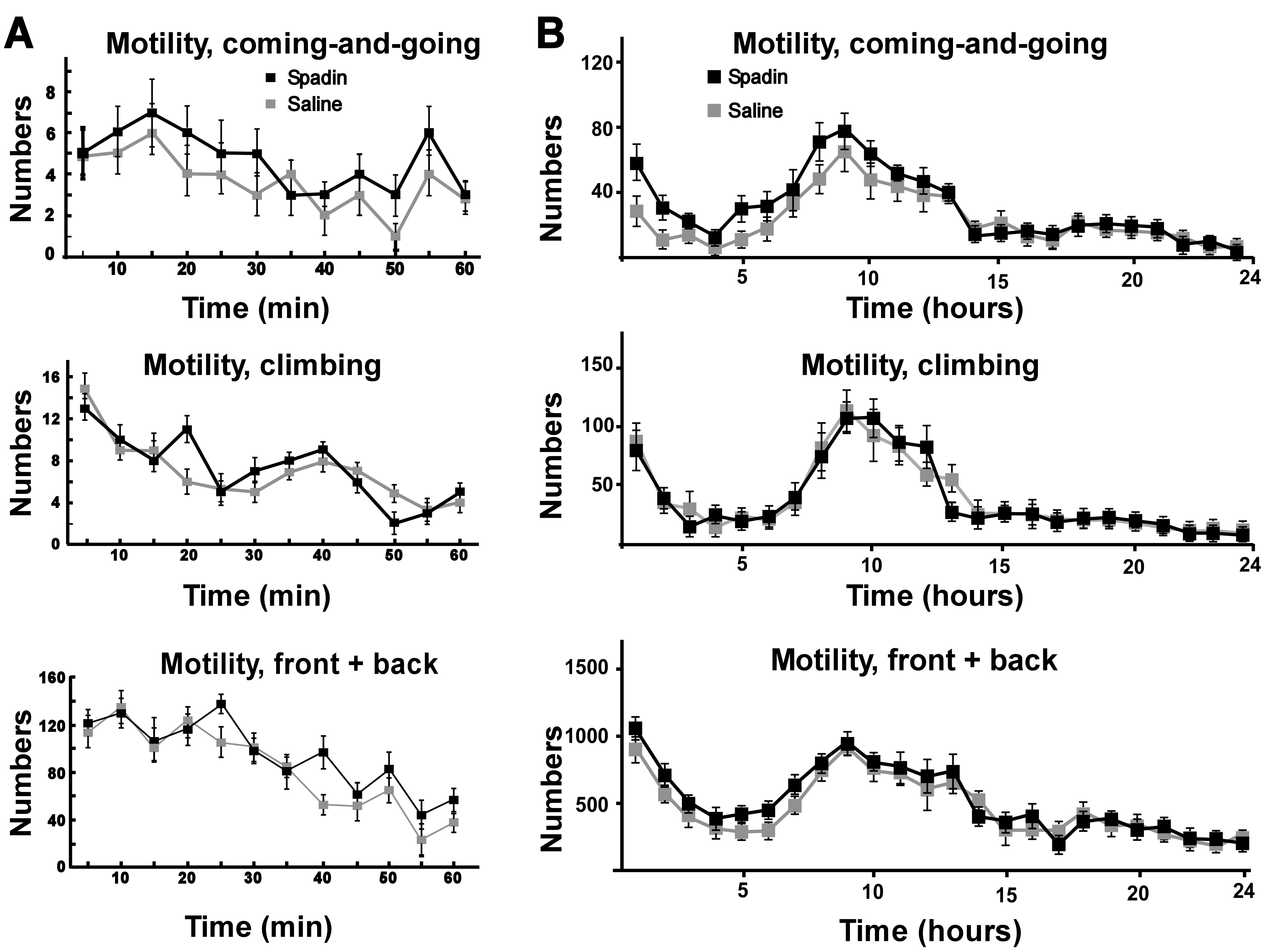

Supplement: Figure S6 — Mouse locomotion activity. To determine whether spadin induced a change in locomotor activity, mice (n = 8 per group) were injected with the saline solution or spadin (10−5M in 100 µl bolus, i.p.) 30 min before starting the test session. Locomotor activity was monitored individually for 24 h using an infrared photobeam activity monitoring system (Imetronic, Pessac, France), which measured consecutive horizontal beam breaks. Testing was in transparent plastic cages (43×2 0×20 cm3) with fresh bedding in a grid of 8 cm horizontal infrared beams. Locomotor activity was defined as breaking of consecutive photobeams. Mice were given 1 h habituation session before being treated with spadin or saline. Movements were recorded and totalized for each 5 min period during the first hour and then by 10 min time sections for the next 23 h. Six periods were pooled to obtain data for 1 h of time. Different movements were monitored: the coming-and-going between the back and the front of the cage, climbing, and other movements in the back or the front of the cage. Data are the mean value of 8 animals per condition, bars represent SEM. Mice were kept under standard laboratory conditions: 12∶12 light-dark cycle with free access to food and water during the experiment. There was no significant difference in locomotor activity between spadin- and saline-treated mice within 1 or 24 h after the drug injection. (0.53 MB TIF) [file pbio.1000355.s006.tif]
