# Supplementary material for: Spadin, a Sortilin-Derived Peptide, Targeting Rodent TREK-1 Channels: A New Concept in the Antidepressant Drug Design
Source: PLoS Biol. 2010 Apr 13;8(4):e1000355. doi: 10.1371/journal.pbio.1000355 (PMC2854129; doi:10.1371/journal.pbio.1000355)
Supplement: Text S1 — Supplementary material and methods. (0.07 MB DOC) [file pbio.1000355.s007.doc]

**Materials and Methods**

***Generation of TREK-1 deficient mice [1].*** TREK-1 genomic clones were isolated from a 129 mouse genomic library by using a *TREK-1* cDNA probe and subcloned into pBluescript SK (Stratagene). The Floxed targeting vector was generated from a 7.5 kb Bgl2/EcoR1 restriction fragment containing exon 1-3 of the *KCNK2* gene. The vector was designed to allow CRE-mediated deletion of exon 3 which encodes the TM1 domain of the channel. A first loxp sequence was inserted in the 5’ flanking intron of exon 3. Similarly, the PGK-neomycin resistance cassette (neo) was inserted together with a second loxp sequence in the 3’ flanking intron of exon 3. Both loxp sequences were in the same orientation to allow CRE‑mediated simultaneous excision of Exon 3 and neo cassette. A copy of the diphteric toxin gene was subcloned adjacent to the homologous region for negative selection of the ES clone. The targeting vector (50μg) was linearized prior to electroporation into 129‑derived embryonic stem cells. After drug selection (G-418, 350μg/ml), one positive clone (1/288) was identified by Southern blot and PCR analysis. Five highly chimeric males were generated by injection of the targeted ES cells into C57Bl/6J blastocysts. 8-10 weeks old male kcnk2*^+/+^* and kcnk2*^-/-^* used for behavioral experiments were from N11 C57Bl/6J derived ^+/-^F4 intercross littermates. kcnk2*^+/-^* (N11 backcross to C57Bl/6J) were crossed to generate ^+/+^ and ^-/-^ male and female littermates (N11F4). Male and female of the same genotype (either ^+/+^ or ^-/-^) were then crossed to each other to generate large number of animals used for behavioral experiments. For pharmacological studies, we used C57Bl/6J mice as wild-type controls. Pilot studies have shown that the results arising from behavioral experiments performed on littermate’s N11F4 kcnk2^+/+^ and C57Bl/6J mice were comparable (data not shown). It is the reason why we considered C57Bl/6J mice as wild-type mice.

***Rat extracellular unitary recordings of DRN 5-HT neurons.***

Single-barreled glass micropipettes (recording electrodes) were filled with a 2 M NaCl solution saturated with Fast Green FCF, resulting in an impedance of 2–5 MΩ. Rats were anaesthetized with chloral hydrate (400 mg/kg, *i.p.*, using a 8% solution), and placed in a stereotaxic frame. A burr hole was drilled on the midline 1 mm anterior to lambda. DRN 5-HT neurons were encountered over a distance of 1 mm starting immediately below the ventral border of the Sylvius aqueduct. They were identified using the following criteria: a slow (0.5–2.5 Hz) and regular firing rate and long-duration (0.8–1.2 ms) action potentials, with a positive-negative spike [2](Lucas and Debonnel, 2002). Spikes were computed by using the Spike 2 software, so that the firing rate was calculated as the mean number of events occurring within a 10 s period. For each neuron, the discharge was monitored during 60 seconds. Each rat received either spadin (10^-5^M in a 500µl bolus) or its vehicle. Starting 30 min after the injection, 2 to 3 successive descents were performed along the DRN, for a total of 8-12 cells recorded per animal. Recordings were performed for a maximal duration of 4 hours post-injection.

***Behavioral tests***

The behavioral experiments were performed blind to experimental groups and genotype in a quiet room by the same investigator for a given test. All mice were naïve in each behavioral test used.

***Depression behavioral tests***

***Porsolt forced swim test (FST) [3].*** Mice (Janvier, France) were placed individually in cylinders (height: 30 cm, diameter: 15 cm) filled with 12 cm deep water (temperature: 22 ±1°C) for 6 min. The total period of immobility was recorded during the last 4 min.

Male Sprague-Dawley rats (Janvier, France), weighing 250-300 g were also used for the FST. Rats experienced a pretest session followed 24 h later by a test session. For both the pretest and the test sessions, conducted under low illumination (15 W), the animals were placed in a plastic cylindrical tank (50 cm high by 20 cm in diameter) filled with 40 cm deep water at 24°C. The pretest was carried out for 15 minutes and the test for 5 minutes in the same tank. The total period of immobility was analyzed during the last 4 min.

***Tail suspension test (TST) [4]*.** Mice were suspended by the tail. After an “agitation” or “escape-like” behavior, mice adopted an immobile posture, suggested to mirror a state of depression. The immobility time was recorded during a 6 min test session.

***Conditioned Suppression of Motility (CSM) test [5].*** Mice were placed in a rectangular cage (20 x 20 x 27 cm) with a metallic grid floor, located in a sound-proof room illuminated by a 75W bulb. There were 12 squares drawn on the floor for counting animal displacements. On the first day, the mouse was left in the test cage for 6 min and received 30 electric footshocks of 1.8 mA during 200 ms (one shock every 12 s.). On the second day, the mouse was again placed in the same cage without receiving electric footshocks (conditioned suppression group). Motility changes were observed by counting the number of squares crossed plus the number of climbing in 6 min. Each control group was treated exactly by the same way, except for the absence of the electric foot-shock. In this test, the mice learned to associate a conditioned stimulus (CS: test chamber) with an unconditioned stimulus (US: footshocks). After the pairing of CS and US, a robust associative memory of the CS-US was formed such that the CS alone could elicit a fear response (behavioral immobility or freezing).

***Learned helplessness (LH) test [6].*** LH training was carried out in 2-way shuttle plexiglas boxes (Imetronic). Each box was divided in 2 equal compartments (19.5 x 10 x 13 cm) separated by a wall equipped with a gate (4.7 x 4.7 cm) that was closed during LH training but opened automatically during shuttle escape testing. LH was induced in shocked group by administering 360 inescapable, 2-s duration footshocks (0.3 mA intensity), administered once every 10 s over a 1-hour session. Scrambled shocks were delivered by a source shock to a grid floor, which was made of stainless steel bars 2 mm in diameter, spaced 4 mm apart. A non-shocked control group was exposed to the apparatus for an equivalent period of time but did not received shock. 24 hours later, escape testing was performed in the same box, but the animals had the possibility to avoid shocks by moving through the gate to the other side of the box. During testing sessions, all mice were given 30 shuttle escape trials with 30-s intervals between the start of each trial. While on the first five trials, the gate opened at the same time as the shock (0.3 mA), for the 25 remaining trials, the gate opened 2 s after shock onset. Each trial (with a maximal 24-s) duration was terminated when the mouse went into the adjacent compartment. Escape latencies were averaged over 5 trials. Overall escape latencies were computed by averaging escape latencies over 30 trials.

***Novelty–suppressed feeding test (NSF) [7]***. NSF paradigm is a conflict test that elicits competing motivations: the drive to eat and the fear of venturing into the center of a bright arena. Latency to begin eating is used as an index of anxiety- and depression- like behavior. After 4 day treatment (saline or spadin, *i.v.*) and 24 hours food-deprivation, mice were presented with a food pellet placed in the center of a brightly plastic box (50 x 50 x 20 cm). Latency to begin eating was measured as previously described [7, 8].

***Anxiety behavioral tests***

***Elevated plus-maze [9].*** The apparatus consisted of two open and two enclosed arms of the same size (45x5 cm) with walls 15 cm high. The arms, constructed of black acrylic, radiate from a common central platform (5x5 cm) to form a plus sign. Open arms were arranged opposite to one another, as were closed arms. The maze was elevated to a height of 45 cm above floor level. Each mouse was placed in the central platform facing one of the open arms. The number of entries into the open and closed arms and the time spent on the open and closed arms were recorded during a 5 min test period. Time spent in the unprotected open arms as well as the number of entries in open arms in relation to the total of arm entries are used as experimental indices of anxiety.

***Light/dark exploration [10].*** The apparatus consisted of a cage (25X40X20 cm) divided into two compartments by a black partition containing a small opening that allows mouse to move from one compartment to the other. One compartment, comprising one-third of the surface area, was made of white plastic and was brightly illuminated. The adjoining smaller compartment was black and dark. Mice were placed in the white compartment and allowed to move freely between the two chambers for 5 minutes. The number of transitions between the two compartments, time spent in the white chamber, and latency to the first transition were recorded. Mice tend to avoid the white compartment. Thus the measures of exploration in this area (time and entrances) were used as experimental indices of anxiety.

***Stair case test [11]***. The apparatus comprised an enclosed staircase consisted of five identical steps (2.5 cm in height X 11 cm in width X 7.5 cm in length), each made of black plastic. The height of the walls was constant (12.5 cm above the stairs) along the entire length of the staircase. Each mouse was placed individually on the floor of the staircase with its back to the staircase. A step was considered climbed only if the mouse placed all four paws on it. Rearing was recorded when the mouse rose on its hind legs either on the step or against the wall. The number of steps ascended and the number of rearings made in a 3-min period were recorded. Results were expressed as ratio of rearing/step numbers.

**Statistical Analyses.**

Data were expressed as mean ± S.E.M. Statistical analysis of differences between groups was performed by using unpaired *t* test or ANOVA (with one or two factors and with or without repeated measured where appropriate). Where F ratios were significant, statistical analyses were extended and post-hoc comparisons made by using LSD, Newman-Keuls or Tukey’s test multiple comparison tests. The level of significance was set at *P* < 0.05.

**REFERENCES**

1. Heurteaux, C., et al. (2004) TREK-1, a K(+) channel involved in neuroprotection and general anesthesia. Embo J 23: 2684-95.

2. Lucas G, Debonnel G (2002) 5-HT4 receptors exert a frequency-related facilitatory control on dorsal raphe nucleus 5-HT neuronal activity. Eur J Neurosci 16: 817-822.

3. Porsolt RD, Le Pichon M, Jalfre M (1977) Depression: a new animal model sensitive to antidepressant treatments. Nature 266: 730-732.

4. Steru L, Chermat R, Thierry B, Simon P (1985) The tail suspension test: a new method for screening antidepressants in mice. Psychopharmacology (Berl) 85: 367-370.

5. Kameyama T, Nagasaka M, Yamada K (1985) Effects of antidepressant drugs on a quickly-learned conditioned-suppression response in mice. Neuropharmacology 24: 285-290.

6. Caldarone BJ, George TP, Zachariou V, Picciotto MR (2000) Gender differences in learned helplessness behavior are influenced by genetic background. Pharmacol Biochem Behav 66: 811-817.

7. Santarelli L, Saxe M, Gross C, Surget A, Battaglia F, et al. (2003) Requirement of hippocampal neurogenesis for the behavioral effects of antidepressants. Science 301: 805-809.

8. Heurteaux C, Lucas G, Guy N, El Yacoubi M, Thümmler S, et al. (2006) Deletion of TREK-1, a background potassium channel, results in a depression-resistant phenotype. Nature Neurosci 9: 1134-1141.

9. Gross, C., et al. (2002) Serotonin1A receptor acts during development to establish normal anxiety-like behaviour in the adult. Nature 416: 396-400.

10. Welch, J.M., et al.( 2007) Cortico-striatal synaptic defects and OCD-like behaviours in Sapap3-mutant mice. Nature 448: 894-900.

11. Cryan, J.F. et al. (2003) Antidepressant and anxiolytic-like effects in mice lacking the group III metabotropic glutamate receptor mGluR7. Eur J Neurosci 117: 2409-2417.
